# Strain-specific responsiveness of hepatitis D virus to interferon-alpha treatment

**DOI:** 10.1016/j.jhepr.2023.100673

**Published:** 2023-01-24

**Authors:** Katja Giersch, Paulina Perez-Gonzalez, Lennart Hendricks, Nora Goldmann, Jonathan Kolbe, Lennart Hermanussen, Jan-Hendrick Bockmann, Tassilo Volz, Annika Volmari, Lena Allweiss, Joerg Petersen, Dieter Glebe, Marc Lütgehetmann, Maura Dandri

**Affiliations:** 1 Department of Internal Medicine, University Medical Center Hamburg-Eppendorf, Hamburg, Germany; 2 Institute of Medical Virology, National Reference Center for Hepatitis B Viruses and Hepatitis D Viruses, Justus Liebig University Giessen, Giessen, Germany; 3 German Center of Infection Research (DZIF), Hamburg-Lübeck-Borstel-Riems and Giessen-Marburg-Langen Partner Sites, Germany; 4 IFI Institute for Interdisciplinary Medicine at Asklepios Clinic St. Georg, Hamburg, Germany; 5 Department of Medical Microbiology, Virology and Hygiene, University Medical Center Hamburg-Eppendorf, Hamburg, Germany

**Keywords:** HDV, Human liver chimeric mice, Resistance, Antiviral, Genotype, Actb, actin beta, ADAR, adenosine deaminase, ADF, adefovir, AG, antigenomic, BSA, bovine serum albumin, casp, caspase, CHD, chronic hepatitis D, CK18, cytokeratin 18, CXCL10, C-X-C motif chemokine ligand 10, Eef2, eukaryotic elongation factor, FCS, foetal calf serum, GAPDH, glyceraldehyde-3-phosphate dehydrogenase, hAAT, human alpha antitrypsin, HBsAg, hepatitis B virus surface antigen, HDAg, hepatitis delta antigen (S, small, L, large), HLA, human leucocyte antigen, HSA, uman serum albumin, IFNα, interferon α, ISGs, interferon stimulated genes, LAM, lamivudine, LLoD, lower limit of detection, Mavs, mitochondrial antiviral-signalling protein, MDA5, melanoma differentiation-associated protein 5, MoA, mode of action, MOI, multiplicity of infection, MxA, myxovirus resistance gene A, NTCP, sodium (Na+) taurocholate co-transporting polypeptide, NUCs, nucleos(t)ide analogues, OAS1, 2′-5′-oligoadenylatsynthetase 1, PEG, polyethylene glycol, pegIFNα, pegylated interferon alpha, PHHs, primary human hepatocytes, pgRNA, pregenomic RNA, Rig-I, retinoic acid-inducible gene I, RNP, ribonucleoprotein, qPCR, quantitative real time polymerase chain reaction, SCID, severe combined immunodeficiency, STAT1, signal transducers and activators of transcription 1, TGFβ, transforming growth factor-β, uPA, urokinase plasminogen activator, USG, uPA/SCID/beige/IL2RG-/-

## Abstract

**Background & Aims:**

Pegylated interferon alpha (pegIFNα) is commonly used for the treatment of people infected with HDV. However, its mode of action in HDV-infected cells remains elusive and only a minority of people respond to pegIFNα therapy. Herein, we aimed to assess the responsiveness of three different cloned HDV strains to pegIFNα*.* We used a previously cloned HDV genotype 1 strain (dubbed HDV-1a) that appeared insensitive to interferon-α *in vitro*, a new HDV strain (HDV-1p) we isolated from an individual achieving later sustained response to IFNα therapy, and one phylogenetically distant genotype 3 strain (HDV-3).

**Methods:**

PegIFNα was administered to human liver chimeric mice infected with HBV and the different HDV strains or to HBV/HDV infected human hepatocytes isolated from chimeric mice. Virological parameters and host responses were analysed by qPCR, sequencing, immunoblotting, RNA *in situ* hybridisation and immunofluorescence staining.

**Results:**

PegIFNα treatment efficiently reduced HDV RNA viraemia (∼2-log) and intrahepatic HDV markers both in mice infected with HBV/HDV-1p and HBV/HDV-3. In contrast, HDV parameters remained unaffected by pegIFNα treatment both in mice (up to 9 weeks) and in isolated cells infected with HBV/HDV-1a. Notably, HBV viraemia was efficiently lowered (∼2-log) and human interferon-stimulated genes similarly induced in all three HBV/HDV-infected mouse groups receiving pegIFNα. Genome sequencing revealed highly conserved ribozyme and L-hepatitis D antigen post-translational modification sites among all three isolates.

**Conclusions:**

Our comparative study indicates the ability of pegIFNα to lower HDV loads in stably infected human hepatocytes *in vivo* and the existence of complex virus-specific determinants of IFNα responsiveness.

**Impact and implications:**

Understanding factors counteracting HDV infections is paramount to develop curative therapies. We compared the responsiveness of three different cloned HDV strains to pegylated interferon alpha in chronically infected mice. The different responsiveness of these HDV isolates to treatment highlights a previously underestimated heterogeneity among HDV strains.

## Highlights

• Responsiveness of three HDV strains to pegylated interferon alpha (pegIFNα) was tested *in vivo.*
• A newly cloned HDV-1 and an HDV-3 isolate strongly responded to pegIFNα.
• In contrast, a commonly used HDV-1 strain was IFN-resistant *in vivo.*
• PegIFNα reduced HBV in all co-infected mice and induced similar innate responses.
• The study indicates the existence of virus-specific determinants of IFNα responsiveness.

## Graphical abstract

**Figure:**
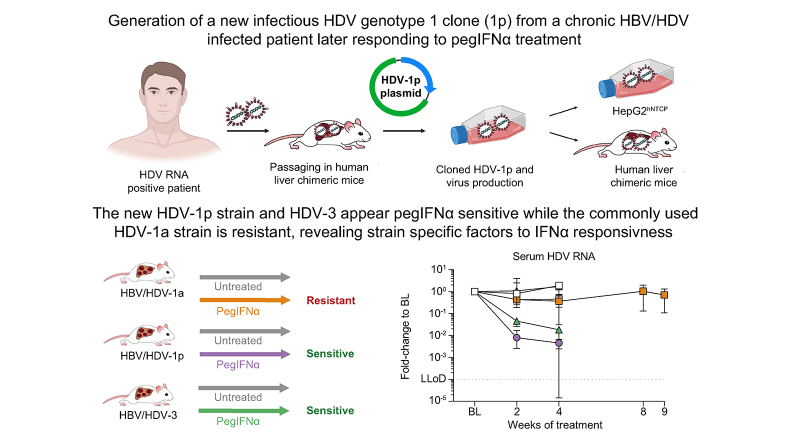

## Introduction

The hepatitis delta virus (HDV) infects around 20 million people worldwide^1^ and recent reports suggested that the number of HDV-positive individuals may be even higher.[1], [2], [3] Liver disease associated with chronic hepatitis D (CHD) causes substantial global morbidity (cirrhosis, hepatocellular carcinoma) and mortality.^4^ HDV RNA replication takes place in the nucleus of hepatocytes by hijacking the host RNA polymerase II, which amplifies the genomic HDV RNA through a double rolling-circle amplification process.^5^ During HDV replication, two additional viral RNAs accumulate in the hepatocytes: the antigenomic RNA, which is an exact complement of the genomic RNA and the smaller linear mRNA encoding for the hepatitis delta antigen (HDAg). HDAg binds specifically to the HDV RNA and exists in two different forms: the small HDAg (S-HDAg) that is important for virus replication and the large variant (L-HDAg), which can inhibit replication and promotes virus assembly through a prenylation site.^5^ The L-HDAg is generated by post-transcriptional RNA editing at the adenosine 1012 (amber/W site), which is mediated by the RNA-specific adenosine deaminase (ADAR). The balance between viral replication and assembly is orchestrated by the presence of S- and L-HDAg and by post-translational modifications of these proteins, such as prenylation, phosphorylation, methylation, and SUMOylation.^6^ HDV is a satellite virus that requires expression of hepatitis B virus (HBV) envelope proteins to release infectious HDV particles.^7^ Both HBV and HDV infect the human hepatocytes via the sodium taurocholate co-transporting polypeptide (NTCP).^8^ Moreover, and in strong contrast to HBV, HDV can also disseminate through cell division.^9^^,^^10^ Active HDV infection either can occur upon simultaneous co-infection with HBV or as a super-infection in people already infected with HBV. HDV is classified into eight genotypes. Although HDV-1 is the most prevalent genotype worldwide, HDV-3 is frequently associated with the most severe hepatitis.^11^^,^^12^ Sequence divergence among the genotypes is as high as 40% over the entire RNA genome with the greatest difference observed between HDV genotype 1 (HDV-1) and genotype 3 (HDV-3).^13^

Because of its compact genomic organisation and lack of its own polymerase, HDV offers very few therapeutic targets and current HBV therapies based on the use of nucleos(t)ide analogues (NUCs) inhibiting the HBV polymerase cannot directly target HDV. Understanding the factors that are able to block HDV infection and replication is therefore of utmost importance for the development of HDV curative therapies. In 2020, the HBV/HDV entry inhibitor bulevirtide (Hepcludex/Myrcludex-B) obtained conditional marketing authorisation by the EMA as the first HDV-specific drug,^14^ and pegylated interferon lambda and the prenylation inhibitor lonafarnib are currently being tested in clinical trials.^15^ Aside such advances, pegylated interferon-alpha (pegIFNα) has been the most commonly used off-label treatment against HDV for decades. However, treatment is associated with substantial side effects and leads to sustained virological response (defined as undetectable serum HDV RNA 6 months after treatment) in only about 25–30% of patients, with high relapse rates after treatment cessation. Nevertheless, various clinical trials are currently evaluating the contribution of IFN treatment to HDV therapy in novel combination regimens.^16^ Apart from acting as an immunomodulatory agent, IFNα induces interferon stimulated genes (ISGs) via the janus kinase-signal transducer and activator of transcription (JAK-STAT) signalling pathway also in hepatocytes, thereby triggering a cellular antiviral state.^17^ Nonetheless, the mode of action (MoA) of IFNα in stably HDV-infected primary hepatocytes remains elusive. HDV is sensed by the pattern recognition receptor melanoma differentiation antigen 5 (MDA5) of the hepatocytes^18^ leading to ISG enhancement and chemokine production in HBV/HDV infected cells.^19^^,^^20^ However, such antiviral state does not limit HDV replication, whereas therapeutically applied more stable (pegylated) IFNs could affect a patient-derived HDV inoculum *in vivo.*^17^ Moreover, IFNα was shown to promote silencing of the HBV genome, thus lowering HBV transcript levels^21^ and to destabilise HDV RNA during cell division.^22^

To date, only a limited amount of HDV strains have been cloned[23], [24], [25], [26], [27] Most studies used a peculiar HDV-1 clone of uncertain human origin^28^^,^^29^ here dubbed HDV-1a (Table 1 and methods), which turned out to be unaffected by IFNα *in vitro*,^18^^,^^30^^,^^31^ leading to the assumption that interferon only marginally impairs HDV RNA replication in stably infected cells.^22^ In this study, we assessed and compared the antiviral and intrinsic host response to pegIFNα treatment *in vivo* using human liver chimeric mice stably infected with HBV and either with HDV-1a or two different human-derived cloned HDV strains: a novel genotype 1 (HDV-1p) and an HDV-3 (Table 1).^32^

**Table 1:** **Characteristics of the source individuals and cloned viruses**.

	HDV-1a	HDV-1p	HDV-3peru
Sex	Male	Male	Male
Age	Unknown	52 years	18 years
Origin	Unclear	Turkey	Peru
Clinical characteristics	Unclear	Chronic HBV and HDV infection	Severe acute HBV and HDV infection
IFNα response	Untreated	Responsive	Untreated
Specifics	Serially passaged in chimpanzees, in a woodchuck, and then cloned	NA treatment when serum was collected; serially passaged in HBV-infected humanised mice and then cloned	Not passaged; initially HBV envelope of genotype F
Accession number	M21012 [29]; AJ00058 [38]^a^	OL825606	L22063
Year virus cloning	1988	2019	1993
HDV plasmid for cloning	HDV trimer in pSVL	HDV tandem dimer in pcDNA3.1(+)	pCMV3-Peru-1.2
HBV envelope-expressing plasmid for cloning	pT7HB2.7 (genotype D)	HBV subgenome in pcDNA3.1(+) (genotype D)	pT7HB2.7 (genotype D)
References	[29,38]	[17,33] and this study	[34]

Summary of information available regarding the three HDV strains used in this study, including known participant characteristics, cloning strategies used, referring accession numbers, and references.

aSequence analyses revealed that this HDV-1 clone shows 99.7% identity with M21012 and 99.5% with AJ00058. IFN, interferon; NA, nucleos(t)ide analogue.

## Participants and methods

### Virus generation

The patient-derived HDV-1 isolate (HDV-1p) was isolated from a male individual with CHD from the university clinic of Hamburg,^33^ passaged in human liver chimeric uPA/SCID/beige/IL2RG^-/-^ (USG) mice, sequenced, cloned as genome-sense tandem dimer in pcDNA3.1(+), and infectious particles were produced in cell culture (Table 1). The HDV-3 isolate (Peru-1) was obtained from a young man from Peru, who developed severe acute hepatitis, which was cloned by Casey *et al.*^32^ The origin of the first HDV clone available in the research community is less clear: it is a genotype 1 strain, individual(s) sera were passaged through various chimpanzees at NIH (J. Taylor, pers. commun.), inoculated in a woodchuck and then cloned (Table 1). For the production of the HDV-1a and HDV-3 strains, the HDV recombinant plasmid pSVL(D3) (kindly provided by J. Taylor, Philadelphia, PA, USA)^28^ and pCMV3-Peru-1.2 (kindly provided by J. Casey, Washington DC, USA)^34^ were used. Infectious HDV-1a, HDV-1p, and HDV-3 particles were generated in HuH7 cells as previously described.^35^ In brief, cells were transfected with equimolar amounts of HDV-1a, HDV-1p, or HDV-3 recombinant plasmids and the HBV envelope-expressing vectors pcDNA3.1(+)^36^ (HDV-1p) or pT7HB2.7^37^ (HDV-1a, HDV-3) encoding the surface proteins of HBV genotype D using Fugene HD Transfection Reagent (Promega, Madison, WI, USA) (Table 1).

### Treatment

*In vivo*, pegIFNα treatment was started when HBV/HDV-1a-, HBV/HDV-1p-, or HBV/HDV-3-infected mice reached stable HBV and HDV viraemia levels. Mice received pegIFNα (Pegasys; Roche, Basel, Switzerland) twice a week subcutaneously (25 ng/g body weight)^17^^,^^21^ for 4 or 9 weeks. Mice were sacrificed 24 h after the last pegIFNα injection. Cultured primary human hepatocytes (PHHs) (isolated from an HBV/HDV-infected mouse) received IFNα (1,000 IU/ml; Roferon-A; Roche, Basel, Switzerland) starting 1 day after plating. The culture medium was changed twice a week.

More methods (*e.g.* generation and infection of mice, virological measurements, RNA *in situ* hybridisation) can be found in the supplementary information.

## Results

### Production and infectivity of HDV-1p *in vitro* and *in vivo*

**Fig. 1:**
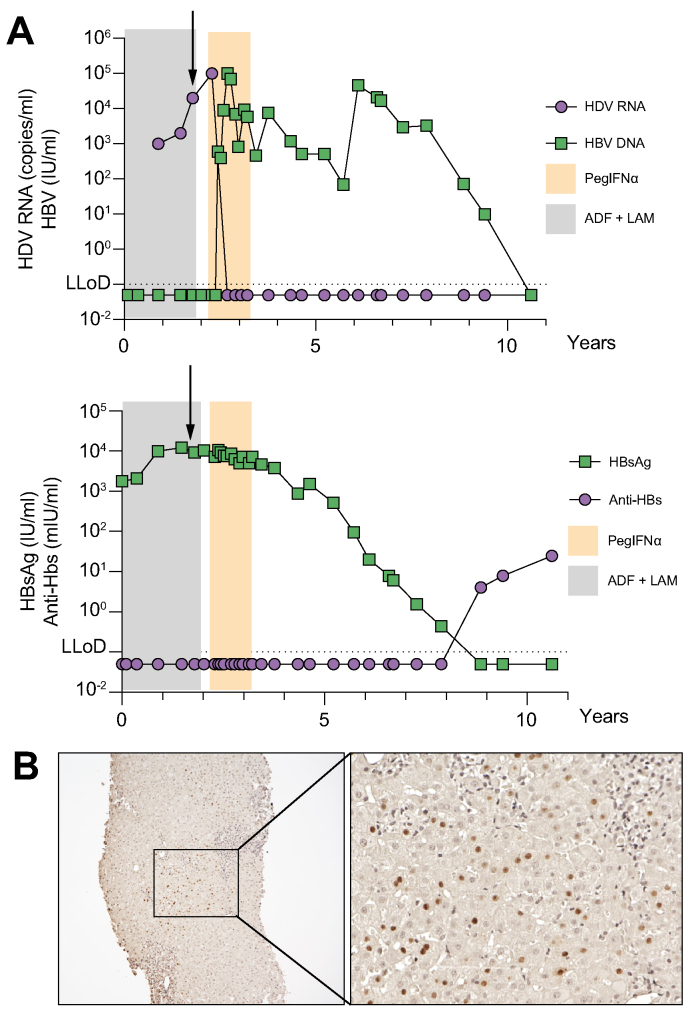
Participant characteristics (HDV-1p). (A) HDV and HBV viraemia (above) and HBsAg and anti-HBs levels (below) during and after NUC (grey box) and pegIFNα treatment (orange box) of the infected individual. The black arrow indicates the time point when serum was collected to infect human liver chimeric mice for passaging the virus. (B) Immunohistology of HDAg (brown nuclei) in a liver biopsy of the individual before treatment with pegIFNα (overview, 10× and close up, 20× ). ADF, adefovir; anti-HBs, hepatitis B surface antibody; hAAT, human alpha antitrypsin; HDAg, hepatitis D antigen; HBsAg, hepatitis B virus surface antigen; LAM, lamivudine; pegIFNα, pegylated interferon alpha.

As summarised in Table 1, the HDV-1p strain was obtained from a person with CHD receiving nucleos(t)ide analogues (NUCs) treatment (lamivudine plus adefovir), which resulted in undetectable serum HBV DNA levels (Fig. 1A). HDV viraemia was detectable and immunohistology confirmed an abundance of HDAg-positive hepatocytes (Fig. 1A and C). Eventually, after discontinuation of NUC treatment, the individual received pegIFNα for 48 weeks, which resulted in a sustained HDV response and temporary increase of HBV viraemia,^33^ but led to HBV and HBsAg loss and seroconversion to hepatitis B surface antibody years later (Fig. 1B).

**Fig. 2:**
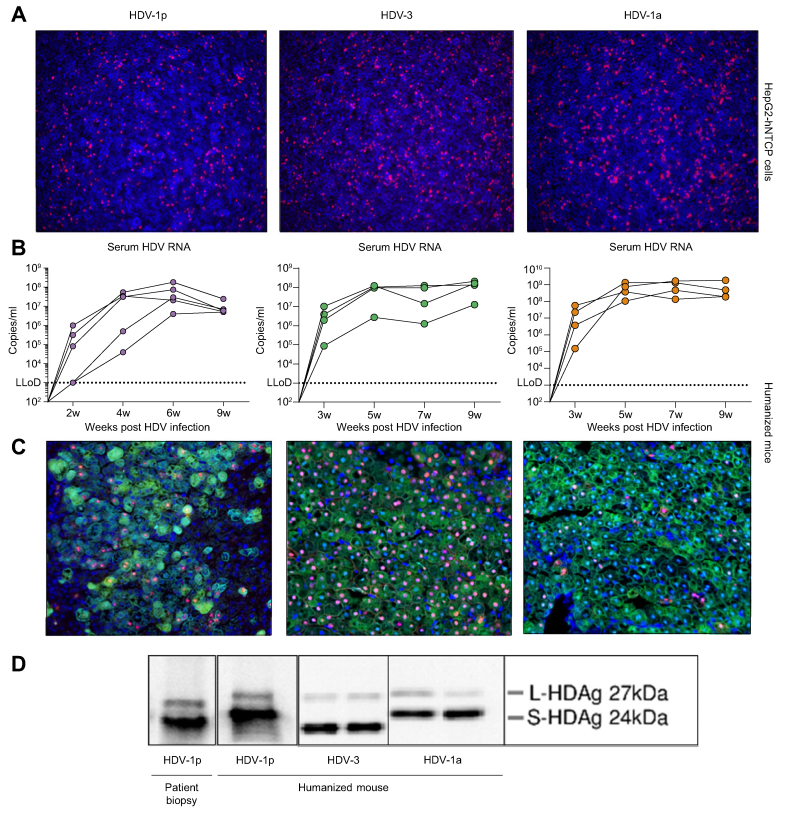
*In vitro* and *in vivo* infectivity of the cloned strains HDV-1p, HDV-3, and HDV-1a. (A) HDAg (red, immunofluorescence [IF] staining) in HepG2^hNTCP^ cells 7 days after mono-infection with HDV-1p, HDV-3, or HDV-1a. (B) Development of HDV viraemia in humanised mice (HDV-1p left, HDV-3 middle, HDV-1a right). IF staining of HDAg (red), HBcAg (green), and the human marker CK18 (turquoise) (C) and Western blot analysis of S- and L-HDAg (D) in stable HBV/HDV-infected mice and in the liver biopsy obtained from the individuals with HDV-1p infection. HDAg, hepatitis D antigen.

The HDV-positive isolate obtained before IFN treatment was first shown to be infectious in HBV-infected human liver chimeric USG mice^17^ and now sequenced and cloned as described in the Participants and methods section (Table 1). The full genome sequence of the HDV-1p strain is available at NCBI (accession number: OL825606). HDV-1p virus stocks were produced in HuH7 cells and their infectivity was tested first *in vitro* using HepG2^hNTCP^ cells. Seven days after HDV-1p inoculation (multiplicity of infection; MOI = 1), HDAg staining (Fig. 2A) confirmed *in vitro* infectivity of the HDV-1p particles produced after cloning the virus. The *in vitro* infectivity of HDV-1p appeared similar to the cloned strains HDV-3 (MOI = 1) and HDV-1a (MOI = 2) (Fig. 2A).

To assess the infectivity of the cloned HDV-1p strain *in vivo*, HBV-infected humanised USG mice were super-infected with HDV-1p from the supernatant of HuH7 cells (4 × 10^6^ GE/mouse). In these HBV infected mice, where HDV infection is first established in a low number of PHHs, HDV disseminated among PHHs and viraemia increased up to week 6 after super-infection and remained stable until the end of the observation time (9 weeks post super-infection) (Fig. 2B). In line with previous studies^39^^,^^40^ we observed a decrease of HBV viraemia (fivefold) during HDV-1p super-infection (data not shown), while HDAg was clearly detected in human hepatocytes (Fig. 2C). Moreover, amounts and distribution of S- and L-HDAg resembled those detected in the patient liver biopsy (Fig. 2D). Likewise, HDV-3 and HDV-1a reached stable HDV titres after around 5 weeks of HDV super-infection and showed similar ratios of S- and L-HDAg (Fig. 2B and D). The amount of HDV-infected human hepatocytes determined by immunofluorescence tended to be higher in mice infected with HDV-3 (63%) compared with those infected with HDV-1a (32%) or HDV-1p (42%), suggesting that HDV-3 may display superior spreading capacities *in vivo* than the HDV-1 strains. Overall, these comparative analyses show that human liver chimeric mice are well suited to study patient-derived viral strains.

### PegIFNα treatment in mice infected with different HDV strains

**Fig. 3:**
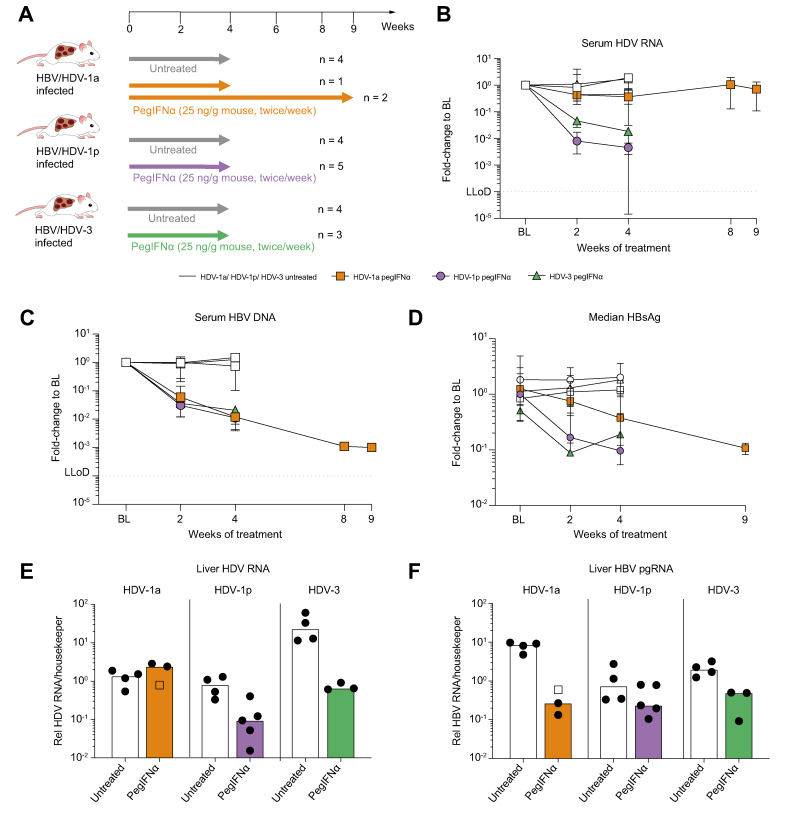
PegIFNα treatment in HBV/HDV-1a, HBV/HDV-1p, and HBV/HDV-3 infected mice. (A) Experimental design: stable HBV/HDV-1a-, HBV/HDV-1p-, and HBV/HDV-3-infected mice received pegIFNα for 4 weeks or remained untreated. Some mice infected with HBV/HDV-1a were treated for 9 weeks. Serum HDV (B), HBV (C), and HBsAg (D) are depicted as fold change from baseline in HBV/HDV-1a-infected untreated (grey line, square symbols; n = 4) or treated mice (blue line, square symbols; n = 3), in HBV/HDV-1p-infected untreated (grey line, round symbols; n = 4) or treated mice (pink line, round symbols; n = 5) and in HBV/HDV-3-infected untreated (grey line, triangular symbols; n = 4) or treated mice (light red line, triangular symbols; n = 5). Curves show median levels with range. PCR measurements of liver HDV RNA (E) and HBV pgRNA (F) in untreated and treated HBV/HDV-1a, HBV/HDV-1p, and HBV/HDV-3 infected mice. Bars show median levels black dots represent individual mice. HBV/HDV-1a infected mice are shown as clear square or black dots when they were treated for 4 (n = 1) or 9 weeks (n = 2), respectively. BL, baseline; HBsAg, hepatitis B virus surface antigen; LLoD, lower limit of detection; pegIFNα, pegylated interferon alpha.

Human liver chimeric mice stably infected with HBV and one of the three HDV clones received pegIFNα twice a week (Fig. 3A). In HBV/HDV-1p and HBV/HDV-3 infected mice, 4-week pegIFNα treatment reduced HDV viraemia by more than 2.0-log and by 1.7-log, HBV viraemia by 2.0-log and 1.7-log, respectively, as well as levels of circulating HBsAg (Fig. 3B–D, and Fig. S1A–D). Intrahepatic analyses revealed a clear reduction of HDV RNA (HDV-1p: 0.9-log, HDV-3: 1.5-log) (Fig. 3E) and HBV pgRNA levels relative to human hepatocyte contents (Fig. 3F) in mice infected with HBV/HDV-1p (0.5-log) or HBV/HDV-3 (0.6-log) in comparison with untreated control mice. In striking contrast, 4-week treatment of HBV/HDV-1a infected mice clearly reduced HBV DNA (2-log) and HBsAg in serum (Fig. 3C and D and Fig. S1B); intrahepatic pgRNA (1.5-log) (Fig. 3E), but had no effect on HDV in serum (Fig. 3B and Fig. S1E) and liver (Fig. 3E). Even after extending pegIFNα treatment to 9 weeks, serum HDV RNA levels remained comparable in treated and untreated mice harbouring the HDV-1a strain (Fig. 3B and Fig. S1E), while HBV viraemia (3-log) and HBsAg levels decreased even further (Fig. 3C and D, Fig. S1F).

**Fig. 4:**
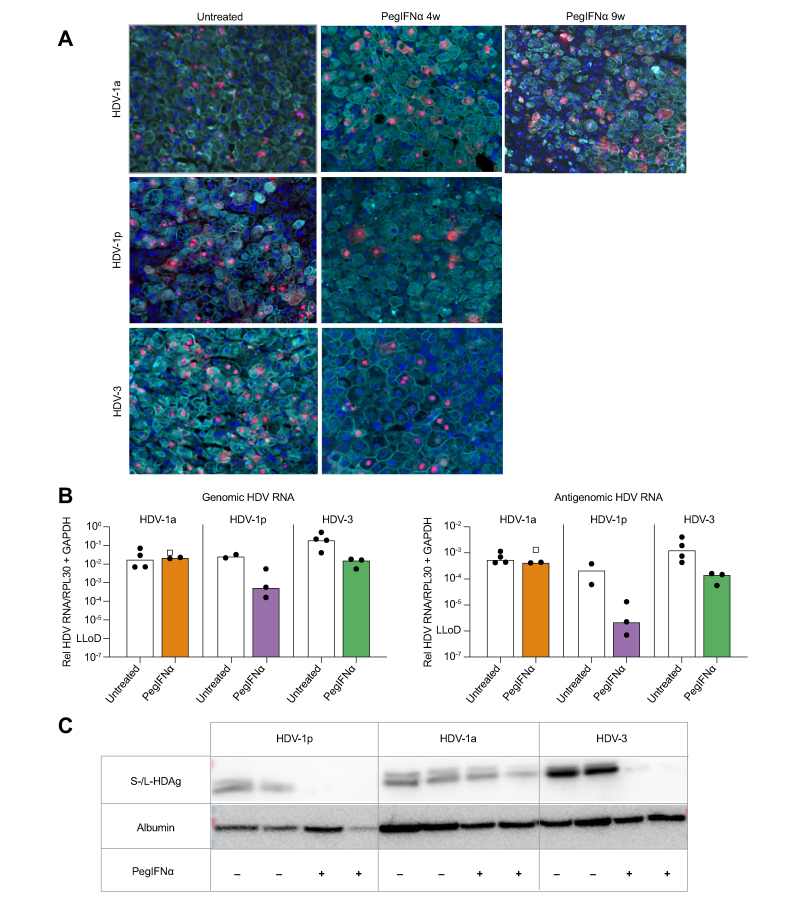
Immunofluorescence staining, genomic/antigenomic HDV RNA and S-/L-HDAg. **(**A) Immunofluorescence staining of HDAg (red) and CK18 (human hepatocytes, aqua) in mouse livers of all groups. Nuclei are stained with Hoechst 33258 (blue). (B) Genomic (left) and antigenomic HDV RNA (right) (qPCR assay using biotinylated magnetic beads) in untreated and treated mice infected with HBV/HDV-1a, HBV/HDV-1p, and HBV/HDV-3. HBV/HDV-1a-infected mice are shown as clear square or black dots when they were treated for 4 (n = 1) or 9 weeks (n = 2), respectively. (C) Western blot analysis of S-HDAg (23 kDa) and L-HDAg (27 kDa) in livers of two untreated and two treated mice (4 weeks) infected with either HBV/HDV-1a, HBV/HDV-1p, or HBV/HDV-3. The amount of human hepatocytes per liver specimen used to extract proteins was estimated by detecting human albumin (67 kDa). GAPDH, glyceraldehyde-3-phosphate dehydrogenase; HDAg, hepatitis D antigen; pegIFNα, pegylated interferon alpha.

In line with liver HDV RNA levels, immunofluorescence staining of HDV-1p- and HDV-3-infected mice 4 weeks after pegIFNα treatment demonstrated lower numbers of HDAg-positive PHHs (HDV-1p: 4%, HDV-3: 30%) compared with untreated controls (HDV-1p: 42%, HDV-3: 63%) (Fig. 4A). This confirms that both HDV-1p and HDV-3 isolates are highly responsive to IFN treatment *in vivo* in mice stably infected with both HBV and HDV. In mice infected with the IFN-resistant HDV-1a strain, the amount of HDAg-positive PHHs appeared comparable between untreated controls (32%) and mice that received pegIFNα for 4 (33%) or 9 weeks (31%) (Fig. 4A).

RNA *in situ* hybridisation showed a clear reduction of genomic HDV RNA in treated HDV-1p and HDV-3 infected mice, while the amount of genomic HDV RNA positive PHHs remained similar in untreated and treated HDV-1a infected mouse livers (Fig. S2). Moreover, genomic and antigenomic HDV RNA levels determined by a strain-specific qPCR assay using biotinylated magnetic beads^17^ revealed that pegIFNα reduced both HDV RNA forms in HDV-1p- and HDV-3-infected mice, but mice harbouring HDV-1a remained unaffected (Fig. 4B). Accordingly, levels of S- and L-HDAg decreased in HDV-1p and HDV-3 infected mouse livers receiving pegIFNα administration but remained comparable in untreated and treated HDV-1a infected mice (Fig. 4C).

### IFNα responsiveness of HDV-1a and HDV-1p *in vitro* using PHHs isolated from infected mice

**Fig. 5:**
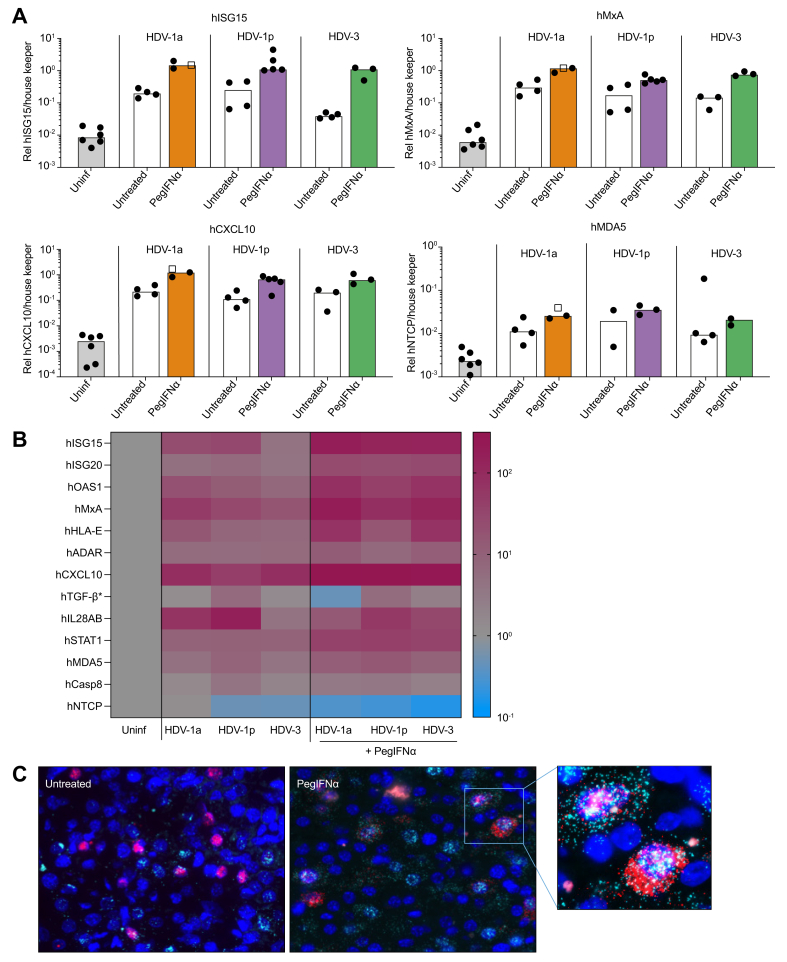
PegIFNα-mediated hISG induction in HDV-1a, HBV/HDV-1p, and HBV/HDV-3 infected mice. (A) qPCR measurements of intrahepatic human hISG15, hMxA, hCXCL10, and hMDA5 mRNA levels in pegIFNα-treated or untreated infected mice compared with uninfected mice (n = 6). Bars show median level, black dots represent individual mice. HBV/HDV-1a-infected mice are shown as clear square or black dots when they were treated for 4 (n = 1) or 9 weeks (n = 2), respectively. (B) The heat map shows mRNA expression levels of different genes in untreated and treated mice of all groups as log induction compared with uninfected controls. Baseline levels are grey, inductions red, and reductions blue. (C) RNA *in situ* hybridisation (RNAScope) staining of antigenomic HDV RNA (red) and hMxA (aqua) in HBV/HDV-1a infected mice. Nuclei are stained with DAPI (blue). CXCL10, C-X-C motif chemokine ligand 10; ISG, interferon stimulated gene; MDA5, melanoma differentiation-associated protein 5; MxA, myxovirus resistance gene A; pegIFNα, pegylated interferon alpha;

To further assess the different responsiveness of HDV-1a and HDV-1p to IFNα in a different experimental setting, PHHs were isolated from humanised mice that were either stably HBV/HDV-1a infected (1.8 × 10^8^ HBV DNA copies/ml, 1.1 × 10^7^ HDV RNA copies/ml) or HBV/HDV-1p infected (2.5 × 10^9^ HBV DNA copies/ml, 1.5 × 10^7^ HDV RNA copies/ml). Plated cells received IFNα for 2 weeks (Fig. 5A). In HDV-1p-infected PHHs, HDV RNA levels remained stable over time, whereas 7- and 14-day IFNα treatment decreased intracellular HDV RNA levels by 1.0-log (89%) and 1.6-log (98%), respectively, compared with untreated controls (Fig. 5B). IFNα also lowered HDV RNA levels in cell culture supernatant by 0.7-log (Fig. 5C), intracellular HBV pgRNA levels by 0.3-log (80%) (Fig. 5D). In contrast, but in line with the *in vivo* results, 2-week IFN treatment did not reduce HDV RNA levels in HDV-1a-infected PHHs (Fig. 5B), although intracellular pgRNA was reduced by 0.5-log (65%) (Fig. 5D). The total amount of cells, determined as ng RNA per well, was not substantially altered over time and by treatment (Fig. 5E).

### PegIFNα-mediated human ISG induction in HBV/HDV-1a, HBV/HDV-1p, and HBV/HDV-3-infected mice

**Fig. 6:**
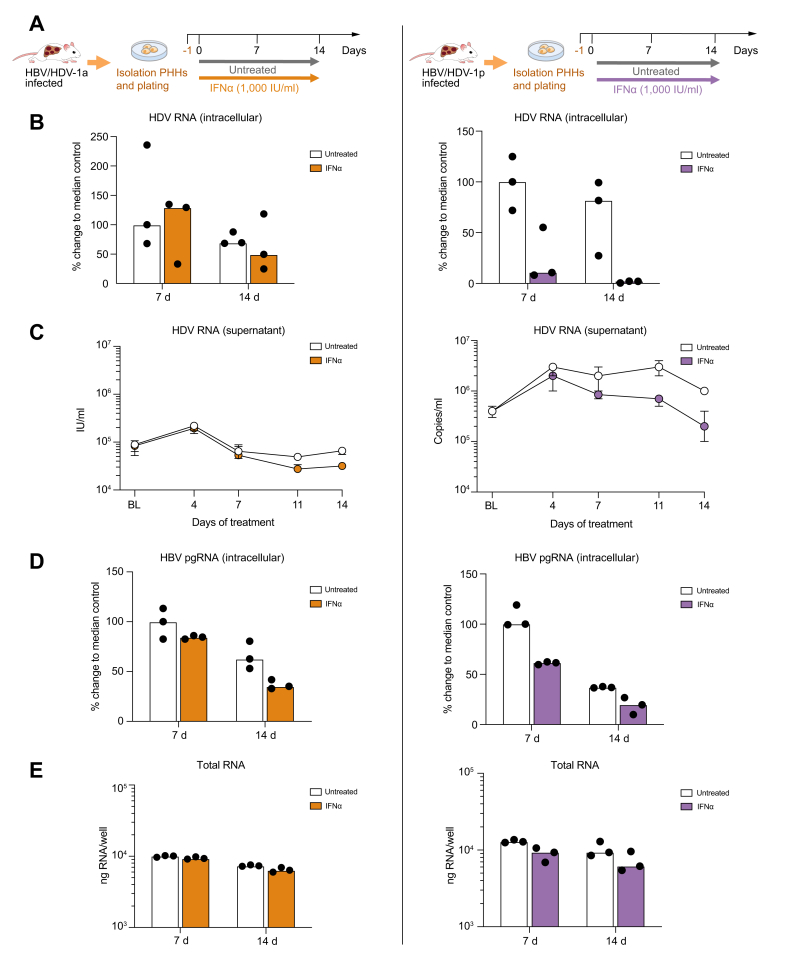
IFNα treatment in HBV/HDV-1a- and HBV/HDV-1p-infected PHHs isolated from humanised mice. (A) Experimental design: PHHs were isolated from a stable HBV/HDV-1a- or HBV/HDV-1p-infected mouse and treated with IFNα. HDV RNA levels in PHHs (percent change from median controls at day 7) (B) and in cell culture supernatant (IU/ml; Baseline, day 4 and 7: n = 6; day 11 and 14: n = 3 for both groups) (C) of infected PHHs 7 or 14 days post IFNα treatment. Percent change of HBV pgRNA levels (D) and the amount of total RNA per well (E) in treated and untreated HBV/HDV-1a or HBV/HDV-1p infected PHHs. Bars show median levels, black dots represent replicates. Blue bars: treated HDV-1a-infected PHHs, pink bars: treated HDV-1p-infected PHHs, grey bars: untreated PHHs. Curves display median levels and range. ADAR, adenosine deaminase; casp, caspase; CXCL10, C-X-C motif chemokine ligand 10; HLA, human leucocyte antigen; IFNα, interferon α; ISGs, interferon stimulated genes; MDA5, melanoma differentiation-associated protein 5; MxA, myxovirus resistance gene A; NTCP, sodium (Na^+^) taurocholate co-transporting polypeptide; OAS1, 2′-5′-oligoadenylatsynthetase 1; PHHs, primary human hepatocytes; STAT1, signal transducers and activators of transcription 1; TGFβ, transforming growth factor-β.

Since mice infected with HBV/HDV-1p or HBV/HDV-3 responded to IFNα treatment and mice infected with HBV and the HDV-1a strain were resistant to therapy, we investigated whether these differences in IFN responsiveness could be explained by the different ability of these viruses to induce intrinsic innate responses in infected hepatocytes. In line with a previous study,^19^ human ISGs (*e.g.* hISG15, hMxA, hOAS1, hSTAT1), pattern recognition receptors (hMDA5), and chemokines (*e.g.* hCXCL10) were similarly and strongly upregulated (between 3- and 88-fold) upon HDV infection, regardless of whether the mice were infected with HBV/HDV-1a, HBV/HDV-1p, or HBV/HDV-3 (Fig. 6A and B, Table 2). PegIFNα treatment induced a further enhancement (between 2- and 29-fold) of human ISGs compared with untreated, infected mice (Fig. 6A and B, Table 2). Interestingly, despite the different HDV treatment outcomes, expression levels of analysed genes appeared comparable among treated animals, suggesting that virological differences, rather than the different enhancement of innate host responses, might be responsible for the antiviral effect of IFN *in vivo*. Furthermore, pegIFNα had no further effect on the expression of human cytokines (hIL28AB, human transforming growth factor-β [hTGF-β]) and on the apoptosis marker human caspase 8, but led to a substantial decrease (3- to 4-fold) of hNTCP expression levels (Fig. 6B, Table 2). Of note, hIL28AB and hTGF-β baseline expression levels are close to the detection limit in PHHs, therefore slight expression changes among groups must be interpreted cautiously.

**Table 2:** **mRNA expression of human genes**.

Gene	Uninfected	HDV-1a		HDV-1p		HDV-3		HDV-1a + pegIFNα		HDV-1p + pegIFNα		HDV-3 + pegIFNα	
	Median rel expr	Median rel expr	Fold ind to uninf	Median rel expr	Fold ind to uninf	Median rel expr	Fold ind to uninf	Median rel expr	Fold ind to uninf	Median rel expr	Fold ind to uninf	Median rel expr	Fold ind to uninf
hISG15	8.70E-03	2.00E-01	23.0	2.55E-01	29.4	4.03E-02	4.6	1.50E+00	7.5	1.11E+00	4.3	1.15E+00	28.5
hISG20	5.72E-03	2.86E-02	5.0	3.55E-02	6.2	2.39E-02	4.2	1.44E-01	5.0	1.28E-01	3.6	1.52E-01	6.4
hOAS1	7.15E-03	1.38E-01	19.3	8.48E-02	11.9	5.08E-02	7.1	5.58E-01	4.0	2.87E-01	3.4	5.04E-01	9.9
hMxA	6.12E-03	3.04E-01	49.7	1.75E-01	28.6	1.06E-01	17.3	1.20E+00	3.9	5.20E-01	3.0	7.80E-01	7.4
hHLA-E	2.02E-02	3.18E-01	15.8	1.61E-01	8.0	1.33E-01	6.6	1.41E+00	4.4	3.30E-01	2.0	1.41E+00	10.6
hADAR	2.51E-02	1.48E-01	5.9	1.41E-01	5.6	1.59E-01	6.3	3.06E-01	2.1	1.62E-01	1.2	2.83E-01	1.8
hCXCL10	2.54E-03	2.23E-01	88.0	1.14E-01	45.0	2.10E-01	82.9	1.25E+00	5.6	6.77E-01	5.9	6.50E-01	3.1
hTGF-β	6.34E-04	7.64E-04	1.2	3.91E-03	6.2	8.81E-04	1.4	2.98E-04	0.4	3.81E-03	1.0	1.55E-03	1.8
hIL28AB	2.55E-05	1.72E-03	67.5	3.93E-03	154.3	1.17E-04	4.6	3.41E-04	0.2	1.38E-03	0.4	7.54E-04	6.5
hSTAT1	1.53E-02	1.38E-01	9.0	1.46E-01	9.5	1.40E-01	9.2	5.86E-01	4.2	6.04E-01	4.1	5.57E-01	4.0
hMDA5	2.35E-03	1.14E-02	4.8	1.98E-02	8.4	9.71E-03	4.1	2.60E-02	2.3	3.58E-02	1.8	2.15E-02	2.2
hCasp8	1.18E-03	1.72E-03	1.5	4.87E-03	4.1	2.26E-03	1.9	3.77E-03	2.2	4.01E-03	0.8	2.77E-03	1.2
hNTCP	3.12E-01	3.61E-01	1.2	1.54E-01	0.5	1.46E-01	0.5	9.89E-02	0.3	8.11E-02	0.5	5.46E-02	0.4

Remarkably, simultaneous visualisation of HDV RNA and human ISGs by RNA ISH revealed that MxA-positive human hepatocytes were still expressing high levels of antigenomic HDV-1a RNA. These results demonstrate that the IFN-resistant HDV-1a isolate does not hamper the IFN-mediated induction of classical human ISGs at the single cell level (Fig. 6C).

### Sequencing of the three distinct HDV genome strains from infected mice

Genome sequencing of intrahepatic HDV RNA in mice infected with HDV-1a, HDV-1p, or HDV-3 revealed that no mutations emerged after 4 or 9 weeks of pegIFNα treatment *in vivo* (data not shown). In addition, the occurrence of genomes encoding for the small (ACC) or large HDAg (ATC) (RNA editing at the amber/W site) remained similar in treated and untreated mice and was comparable between the different HDV isolates (Fig. S3A). The ribozyme site showed 100% identity between the two HDV-1 isolates and 89.6% identity between HDV-1 and HDV-3 (Fig. S3B). The open reading frame for the large HDAg (214 amino acids) showed 89.5% identity between HDV-1p and HDV-1a and 71.2% identity between HDV-1p and HDV-3, which translated into several amino acid changes (Fig. S3B and C). However, the amber/W site (aa196), the prenylation site (aa212), and other known post-translational modification sites remained fully conserved between the three different isolates (Fig. S3C). In line with Le Gal *et al.*,^41^ the two HDV-1 strains showed a proline residue at the nuclear export site at position 205, whereas HDV-3 harbours a glycine, suggesting different virion secretion efficiencies across genotypes. Two unique differences that exclusively occurred in the IFN-resistant HDV-1a-isolate were detected in the coiled coil domain at position aa41 (leucine instead of isoleucine) and aa44 (isoleucine instead of leucine) (Fig. S3C). Although these are conservative mutations, we cannot exclude their impact on SUMOylation rates at position 42.

## Discussion

In CHD, PegIFNα is commonly used as an off-label treatment, although its mode of action in HDV-infected hepatocytes is still unclear and responsiveness to IFN treatment remains limited.^16^ Understanding HDV diversity and the mechanisms determining different antiviral responsiveness to treatment is paramount for improving therapeutic options. Experimental studies with the aim to unravel IFN MoA in HDV infection were hampered not only by the paucity of infection models available, but also by the limited availability of well-characterised HDV isolates. By chance, the first HDV-1 clone that was generated in 1988 (Kuo *et al.*,^29^ herein HDV-1a), appeared IFN-resistant^28^^,^^29^ both in transfected hepatoma cells and infected PHHs.^18^^,^^30^^,^^31^ HDV was reduced only when IFNα was administered around HDV infection time,^18^^,^^30^ indicating that IFNα mainly limited new infection events. These *in vitro* studies led to the general assumption that HDV is resistant to IFN treatment when infection is already established. However, we observed that human liver chimeric mice infected with an HDV-positive patient-derived serum responded to pegIFNα.^17^ Interestingly, this HDV isolate (HDV-1p) was obtained from an individual who later achieved sustained HDV response upon pegIFNα treatment (Fig. 1 and Bockmann *et al.*^33^). To assess the IFN responsiveness of the most commonly used HDV-1a clone *in vivo*, in HBV/HDV infected human hepatocytes, and to compare these data with distinct HDV strains, we cloned this new HDV-1p isolate and also used an additional, genetically distant clone from an HDV-3 strain.^32^

In stable HBV/HDV-1p- and HBV/HDV-3-infected humanised mice, pegIFNα treatment clearly reduced HDV markers in serum and liver, including the levels of genomic and antigenomic HDV RNA, as well as S- and L-HDAg. In striking contrast, we did not detect any antiviral effect on HDV parameters when stable HBV/HDV-1a infected mice received pegIFNα, although all HBV markers, including HBsAg, were clearly reduced. Of note, the IFN dose commonly applied for studies in humanised mice is rather high (adapted to mouse metabolism) and, most importantly, it is given twice per week. Nevertheless, even extending treatment to 9 weeks did not alter HDV RNA levels or the number of HDV-positive human hepatocytes (as determined by immunofluorescence and RNA *in situ* hybridisation) when the HDV-1a strain was used. Notably, HBsAg is needed for the release of HDV particles and the IFN-mediated reduction of HBV serological markers, particularly those linked to cccDNA activity, was shown to be associated with virological responses in people with CHD.^42^ Accordingly, IFN-mediated suppression of HBsAg production should also lower HDV RNA in serum. However, HBV infection and HBsAg levels are generally high in humanised mice and despite the decrease induced by IFN, HBsAg changes were not sufficient to induce substantial decrease of HDV-1a in IFN-treated mice. Our results indicate that also HDV viraemia changes need to be monitored to predict virological responses during IFN treatment.

Consistent with previous *in vitro* studies,^28^^,^^29^ IFNα treatment of PHHs obtained from a stable HBV/HDV-1a-infected mouse had no effect on HDV RNA amounts. However, IFNα led to the reduction of HDV RNA levels in PHHs isolated from a stable HBV/HDV-1p-infected mouse. Because the virus strain was the only variable in these experimental settings (we used the same human hepatocyte donor and HBV inoculum), the sharp differences determined by using different HDV isolates demonstrate for the first time the existence of virus-derived differences. The key IFN-mediated antiviral mechanism acting in stably infected PHHs needs further investigation and the new genotype 1 HDV isolate could also serve for studies aiming at investigating HDV biology, as well as the antiviral activity of new compounds used alone and in combination with IFN.

As we did not measure induction of caspase 8 or detected substantial PHH loss *in vitro* and *in vivo* during treatment, the death of HDV-positive cells unlikely provides the main mechanism of intrahepatic HDV decline. Although we cannot rule out an increase of cellular stress induced both by HDV infection and interferon treatment, the strong HDV decrease determined in mice infected with HDV-1p or HDV-3 rather suggests that IFN inhibited HDV RNA synthesis and/or promoted its destabilisation.

The intrinsic innate responses of the hepatocytes are thought to be central to counteract HDV infection.^18^^,^^20^ To investigate whether these three HDV strains may alter the ability of the human hepatocytes to sense the infection and to respond to IFN administration, we comparatively analysed classical human ISGs in infected and treated humanised mice.^19^ Both the two HDV-1 isolates and the HDV-3 strain similarly enhanced human ISGs, including chemokines and pattern recognition receptors, compared with uninfected control mice. PegIFNα treatment increased the levels of human IFN signalling genes even further and – interestingly – in a comparable manner among all three HDV isolates used, irrespective of their responsiveness to treatment. Even at the single-cell level, HDV-1a-RNA-positive PHHs strongly co-expressed hMxA mRNA, suggesting that this IFN-resistant HDV-1a isolate does not hamper the broad IFN-mediated upregulation of intrinsic innate responses of human hepatocytes *in vivo*. Nevertheless, as we analysed a limited number of genes, we cannot exclude the possibility that other genes may be differentially expressed. Based on these results, it appears that the hepatocyte innate responses cannot be solely responsible for the strikingly different IFN responsiveness of distinct HDV isolates determined in stable HDV-infected cells, again hinting at the existence of virus-specific factors affecting the strength of IFN responsiveness. We did not identify clear mutations in the ribozyme and HDAg-coding region on the HDV genome or at the RNA editing site upon IFN treatment. Interestingly, all known post-translational modification sites in the HDAg open reading frame, including the prenylation site at position 212 and the nuclear localisation signal (aa-66-75) remained fully conserved among these three isolates. The two only differences that exclusively occurred in the IFN-resistant HDV-1a-isolate were detected in the coiled coil domain (leucine instead of isoleucine and *vice versa*) of the HDAg close to a SUMOylation site. However, leucine and isoleucine are both aliphatic, branched hydrophobic amino acids and these mutations may not influence SUMOylation rates at position 42. It remains to be investigated whether more complex sequence and conformational changes residing outside of the more conserved coding regions of HDV account for the intrinsic primary resistance to IFN determined with HDV-1a.

Recently, Zhang *et al.*^43^ treated proliferating HDV-1a-infected hepatoma cells with IFNα and observed a strong block of cell division-mediated HDV spread,^43^ suggesting that HDV RNA molecules can be targeted by intrinsic innate responses preferentially during cell division, a mechanism permitting NTCP-independent HDV spreading among daughter cells.^10^ Experiments in human liver chimeric mice are usually started when human hepatocyte engraftment is completed and cell turnover is low.^9^^,^^10^ Herein, we treated HBV/HDV-infected mice with IFN several weeks after PHH repopulation was accomplished and rates of PHH proliferation did not differ among mice infected with the distinct HDV isolates (Ki67-staining, data not shown). Although additional studies are required to explore the impact of IFN on distinct HDV strains during hepatocyte proliferation, cell turnover appears unlikely to explain the different IFN responses determined in the experimental setting used here. The ability of IFN to limit not only HDV infection events, but also to lower intrahepatic viral loads even in resting hepatocytes, would provide a rationale for the stronger anti-HDV effects determined in people with CHD receiving pegIFNα and the entry inhibitor bulevirtide in combination (MYR-203 clinical trial, NCT02637999).^44^

In conclusion, our study showed that two new patient-derived HDV isolates of genotype 1 and 3 respond to IFNα treatment in immune-deficient human liver chimeric mice. We also provide evidence that the commonly used HDV-1a isolate bears intrinsic capacities to resist IFNα treatment *in vivo*, which were not determined in two other patient-derived isolates. The existence of virus-specific determinants of IFNα responsiveness raises awareness of the need to use different HDV strains to evaluate virological and host-mediated mechanisms of IFN-responsiveness in HDV infection. The availability of new IFN-sensitive HDV strains could also contribute to the development of new therapies aiming at HDV cure.

## Financial support

The study was supported by the 10.13039/501100001659German Research Foundation (DFG) by a grant to MD and ML (SFB 841, A8), and to DG (GL595/9-1 and SFB1021, B08). MD and DG also received funding from the 10.13039/100009139German Center for Infection Research (DZIF-BMBF; TTU-hepatitis 05.820; 05.822; 05.714). The National Reference Center for Hepatitis B Viruses and Hepatitis D Viruses is supported by the German Ministry of Health via the Robert Koch Institute. All funding sources supporting the work are acknowledged and authors have nothing to disclose.

## Authors’ contributions

Initiated and supervised the study: ML, MD. Designed experiments: ML, MD, KG. Generated chimeric mice: AV, LA, TV. Performed analyses and generated data: KG, LHen, PPG, JK, LHer. Performed HDV full genome sequencing: KG. Cloned the HDV-1p and tested their infectivity in HepG2^hNTCP^ cells: NG and DG. Provided clinical data: JHB, JP. Discussed the data: AV, JP, ML. Wrote the manuscript: KG, MD. Corrected the manuscript: NG, DG, AV, LA, PPG.

## Data availability statement

All data, code, and materials used in the analysis are available upon reasonable request for collaborative studies regulated by materials/data transfer agreements (MTA/DTAs) to the corresponding author.

## Conflict of interest

The authors declare no competing interests.

Please refer to the accompanying ICMJE disclosure forms for further details.
