# Supplementary material for: Strain-specific responsiveness of hepatitis D virus to interferon-alpha treatment
Source: JHEP Rep. 2023 Jan 24;5(4):100673. doi: 10.1016/j.jhepr.2023.100673 (PMC9996322; doi:10.1016/j.jhepr.2023.100673)
Supplement: Multimedia component 1 [file mmc1.pdf]

Strain-specific responsiveness of hepatitis D virus to interferon-alpha treatment

Katja Giersch, Paulina Perez-Gonzalez, Lennart Hendricks, Nora Goldmann, Jonathan Kolbe, Lennart Hermanussen, Jan-Hendrick Bockmann, Tassilo Volz, Annika Volmari, Lena Allweiss, Joerg Petersen, Dieter Glebe, Marc Lütgehetmann, Maura Dandri

Table of contents

Supplementary material and methods 2

Table S1 8

Fig. S1 9

Fig. S2. 10

Fig. S3. 11

Supplementary references..**Error! Bookmark not defined.**

Supplementary material and methods

Generation of humanized USG mice. Human liver chimeric urokinase-type plasminogen activator (uPA)/severe combined immunodeficiency (SCID)/ beige/ interleukin-2 receptor gamma chain negative (IL2RG$^{-/-}$) mice (short USG mice) were generated by transplanting one million thawed cryo-preserved human hepatocytes into homozygous USG mice as previously reported [1]. Repopulation rates were estimated by determining human serum albumin (HSA) in mouse sera (ELISA; Bethyl Laboratories, Biomol GmbH, Hamburg, Germany) and human beta-globin in mouse liver DNA (qPCR; Taqman Gene Expression Assay Hs00758889_s1; Applied Biosystems, Carlsbad, USA). Animals displaying high levels of human chimerism (>2 mg/ml HSA in serum) were used for the study. All mice were sacrificed at the end of the experiment (at different time-points as indicated in the results), blood was collected and liver specimens were snap-frozen in chilled isopentane and cryo-conserved at -80°C for further histological and molecular analyses. Mice were maintained under specific pathogen free conditions in accordance with institutional guidelines under approved protocols. All animal experiments were conducted in accordance with the European Communities Council Directive (86/609/EEC) and were approved by the City of Hamburg, Germany.

Cell culture. PHHs were isolated either from one HBV/HDV-1a- or HBV/HDV-1p-infected human liver chimeric mouse and were seeded in 12- or 24-well plates for experiments. PHHs were maintained in William's E medium (Thermo Fisher, Waltham, MA, USA) supplemented with 10% Hyclone FBS (Thermo Fisher, Waltham, MA, USA), 1% GlutaMax (Thermo Fisher, Waltham, MA, USA), 4.7 µg/ml hydrocortisone, 0.1 µg/ml insulin, and 1.8% DMSO. Medium was changed 4 hours after plating and then twice a week. PHHs were maintained in culture for up to 15 days as indicated in the results.

HepG2^{hNTCP} cells were maintained in DMEM medium (Thermo Fisher, Waltham, MA, USA) supplemented with 10% FCS and were seeded in 24-well plates or chamber slides for experiments. Medium was changed twice a week and HepG2^{hNTCP} cells were split weekly (1:6)

before the experiments started. After infection 2% DMSO was added to the medium in order to minimize proliferation of HepG2^{hNTCP} cells. HepG2^{hNTCP} cells were maintained in culture for 7 days as indicated in the results.

Infection. HepG2^{hNTCP} cells were inoculated 24 hours after plating using HDV-1p, HDV-3 or HDV-1a (MOI=1-2) in FCS-free medium containing 4% PEG 8000 and 2% DMSO. 16-24 hours after inoculation the infection medium was removed and cells were washed twice with PBS. Infected cells were maintained until the end of the experiment as described above. Chronic HBV-infected human liver chimeric USG mice (median viremia: $3x10^8$ copies HBV DNA/ml, HBV genotype D) were super-infected with $4x10^6$ HDV genome equivalents per mouse of HDV-1p containing cell culture supernatant. To establish an HBV/HDV-1a or HBV/HDV-3 infection in humanized USG mice, animals were co-infected using HBV (genotype D) and HDV-1a or HDV-3 containing cell culture supernatants (the latest was kindly provided by Camille Sureau, INTS, France) or passaged HBV/HDV-1A positive mouse sera ($1x10^7$ HBV and HDV genome equivalents/mouse). The inoculum corresponded to a MOI of 0.1-0.3 by estimating an average of $3x10^7$ human hepatocytes per mouse liver [2].

Virological measurements in USG mice and PHHs. Viral DNA and RNA was extracted from 200 μl cell culture supernatant or 5 μl mouse serum using the QiAmp MinElute Virus Spin kit (Qiagen, Hilden, Germany) according to the manufacturer's instructions. At the end of the experiments, infected PHHs and mouse livers were collected and intracellular viral DNA and RNA was isolated using the MasterPure™ Complete DNA and RNA Purification Kit (Epicentre, Wisconsin, USA) and the Qiagen RNeasy Mini Kit, respectively.

HDV RNA levels in cell culture supernatant, cells, mouse serum and liver were determined by reverse transcription and qPCR using the ABI Fast 1-Step Virus Master (Applied Biosystems, Foster City, CA, USA) and HDV Taqman primers and probes on an ABI Viia7 (Applied Biosystems) as previously described [3]. HDV-specific primers and probes recognized all HDV genotypes and HDV-1 strains [4]. HDV RNA levels in cell culture supernatant were also

measured using the cobas6800 automated system (Roche, Basel, Switzerland) as previously described [5]. HBV DNA levels in cell culture supernatant and mouse serum as well as HBV pregenomic (pg) RNA levels in cells and mouse liver were determined by qPCR using specific primers and probe (Taqman Gene Expression Assay Pa03453406_s1, Applied Biosystems and [6]) under conditions previously described [3]. Known amounts of an HDV- or HBV-containing plasmid were used as standard for HDV RNA and HBV DNA quantification in serum and cell culture supernatants. Steady-state levels of intracellular viral RNA and DNA amounts were normalized to the median of human specific hGAPDH and hRPL30 (Taqman Gene Expression Assay Hs99999905_m1 and Hs00265497_m1, Applied Biosystems) using the $\Delta\Delta$ct method.

Genomic and antigenomic HDV RNA qPCR assay. Genomic and antigenomic HDV RNA were determined using a biotinylated magnetic beads based qPCR assay as described previously [7]. In brief, RNA extracted from 1 µl mouse liver was reverse transcribed with 0.5 µM of a biotinylated HDV specific forward primer (biotin-GCGCCGGCYGGGCAAC) for genomic HDV RNA or a biotinylated HDV specific reverse primer (biotin-TTCCTCTTCGGGTCGGCATG) for antigenomic HDV RNA detection and the ABI Fast 1-Step Virus Master (Applied Biosystems, Carlsbad, USA). Biotinylated cDNA was purified with the MinElute PCR Purification Kit (Qiagen, Hilden, Germany) and isolated with dynabeads specifically interacting with biotin (Dynal Kilobase Binder Kit, Invitrogen, Darmstadt, Germany) following the manufacturer's instructions. For qPCR 1 µl of purified biotinylated cDNA bound to dynabeads, HDV specific primers and probes [4] and the ABI Fast Advanced Master (Applied Biosystems, Carlsbad, USA) were used. The median of two human-specific housekeeping genes (hGAPDH, Hs99999905_m1, and hRPL30, Hs00265497_m1, Applied Biosystems) were used for normalization.

HDAg Western blot. Western blot of mouse or patient liver tissue was performed as previously described [8]. In brief, protein lysates were obtained by extracting tissue with T-Per Tissue

Protein Extraction Reagent (Pierce, Rockford, United States) supplemented with protease and phosphatase inhibitors. Protein content was measured by Pierce BCA Protein Assay Kit (Thermo Scientific, Rockford, United States). Proteins were resolved on 12% Mini-PROTEAN TGX Precast Gels (Bio-Rad, Feldkirchen, Germany) and blotted on nitrocellulose membranes (0.2 µm pore size; GE Healthcare, Buckinghamshire, UK). S-HDAg and L-HDAg were detected using a rabbit anti-Delta antibody (1:1,000) (kindly provided by John Taylor, Philadelphia, PA USA). Amounts of human hepatocytes were determined by using a mouse anti-human-albumin antibody (1:200,000) (#A6684; Sigma-Aldrich, St. Louis, Missouri, USA). Signals were visualised with Pierce ECL Western Blotting Substrate (Thermo Fisher) and the Fusion FX Imager (Vilber).

Sequencing. For HDV genome sequencing, serum RNA from HBV/HDV-1p- or HDV-1A-infected mice was extracted as described above and cDNA was synthesized with the Transcriptor First Strand cDNA Synthesis Kit (Roche, Basel, Switzerland) using random hexamer primer according to the manufacturer's instructions. To generate the full genome sequence of HDV-1p overlapping PCR fragments were generated using 5 HDV-specific primer pairs [9] and a Red-Taq Polymerase (Sigma-Aldrich, St. Louis, USA) under conditions described previously [9]. To analyze the occurrence of mutations in treated HDV-1p- or HDV-1A-infected mice, the HDV-specific primer pairs R1 and R2 [10] were used as described previously [11]. PCR product length was analyzed on a 0.8% agarose gel and DNA fragments were purified with the MinElute PCR Purification Kit (Qiagen) as recommended by the manufacturer. The forward and reverse strand was sequenced with Sanger sequencing (Mix2seq kit) by Eurofins Genomics (Ebersberg, Germany) and data was analyzed using Geneious R6 (BioMatters, Auckland, New Zealand).

Expression of human interferon stimulated genes (ISGs). To determine intracellular expression levels of human interferon stimulated genes (ISGs) in USG mice, intracellular RNA was extracted as described above and cDNA was synthesized with the Transcriptor First

Strand cDNA Synthesis Kit (Roche, Basel, Switzerland) using oligo-dT primer according to the manufacturer's instructions. qPCR was performed with the ABI Fast Advanced Master (Applied Biosystems) in an ABI Viia7 (Applied Biosystems) and by using Taqman Gene Expression Assays from Applied Biosystems containing human-specific primers and probe, which do not cross-react with murine genes (**suppl. table 1**). The human housekeeping genes hGAPDH (Hs99999905_m1) and hRPL30 (Hs00265497_m1) were used for normalization.

Immune histology. Paraffin-embedded patient biopsies were deparaffinized with xylene, rehydrated with ethanol and boiled in citrate buffer (antigen retrieval). After endogenous protease and protein block biopsies were stained using a rabbit anti-Delta antibody (1:10,000) (kindly provided by John Taylor, Philadelphia, PA USA) at 4 °C overnight. Specific signals were visualized with Dako Envision+ system HRP-labeled polymer anti-rabbit and Dako Liquid DAB+ Substrate Chromogen System (Dako, Glostrup, Denmark). Counterstaining was performed with Mayer's Hematoxylin (Sigma-Aldrich, St. Louis, USA) and slides were dehydrated and mounted. Stained sections were analyzed by brightfield microscopy (Biorevo BZ-9000, Keyence).

Immunofluorescence (IF) staining. Cryostat sections of humanized USG mouse livers were fixed with acetone and stained as previously described [1]. Tissue was incubated with mouse anti-CK18 (1:400, Dako, Glostrup, Denmark), rabbit anti-HBcAg (1:2,000, Dako) and human anti-Delta (anti-HDAg-positive human serum, 1:8,000) primary antibodies at 4 °C overnight. Specific signals were visualized with Alexa 488-, or 555-labeled secondary antibodies (Invitrogen, Darmstadt, Germany). Nuclei were stained with Hoechst 33258 (1:20,000 diluted, Invitrogen). Stained sections were then mounted with fluorescent mounting medium (Dako) and analyzed with the fluorescence microscope BZ8710 (Keyence, Osaka, Japan) using the same settings for the different experimental groups. The percentage of HDAg-positive human hepatocytes was counted manually and by using 3 visual fields (displaying an average of 300-500 human hepatocytes) per mouse liver.

RNA in situ hybridization (RNAScope). RNA *in situ* hybridization was performed on paraformaldehyde-fixed, cryo-preserved mouse liver sections using the RNAScope Fluorescent Multiplex Kit (Advanced Cell Diagnostics, ACD, Hayward, CA, USA) according to the manufacturer's instructions and as previously described [12]. Briefly, liver sections were fixed with 4% paraformaldehyde (PFA), dehydrated with ethanol and pretreated with Pretreat 4 (Pretreatment Kit, ACD) for 30 min. Liver sections were then incubated with RNAScope target probes, which specifically bind HDV-1p genomic (G) HDV RNA (ACD assay number: 484611), HDV-1A genomic (G) HDV RNA (478131), HDV-1A antigenomic (AG) HDV RNA (475311) or hMxA (403831-C3) for 2 h at 40°C (HybEZ oven, ACD). DAPI staining was performed to visualize nuclei. Stained sections were analyzed by fluorescence microscopy (Biorevo BZ-9000, Keyence) using a 60×/1.40 NA oil objective. Merged z stack images were prepared using the same settings for all groups.

Software. Graphs were created with GraphPad Prism (Version 9.3). The graphical abstract was designed with BioRender.

Gene	Assay number (Applied Biosystems)
hISG15	Hs00192713_m1
hISG20	Hs00158122_m1
hOAS1	Hs00973637_m1
hMxA	Hs00895608_m1
hHLA-E	Hs03045171_m1
hADAR	Hs01017595_g1
hCXCL10	Hs00171042_m1
hTGF-β	Hs00171257_m1
hIL28AB	Hs04193049_gH
hSTAT1	Hs01013989_m1
hMDA5	Hs01070332_m1
hCasp8	Hs01018151_m1
hNTCP	Hs00914889_m1

Table S1. Taqman Gene Expression Assays containing human-specific primers and probe. The table shows target genes and assay numbers from Applied Biosystems.

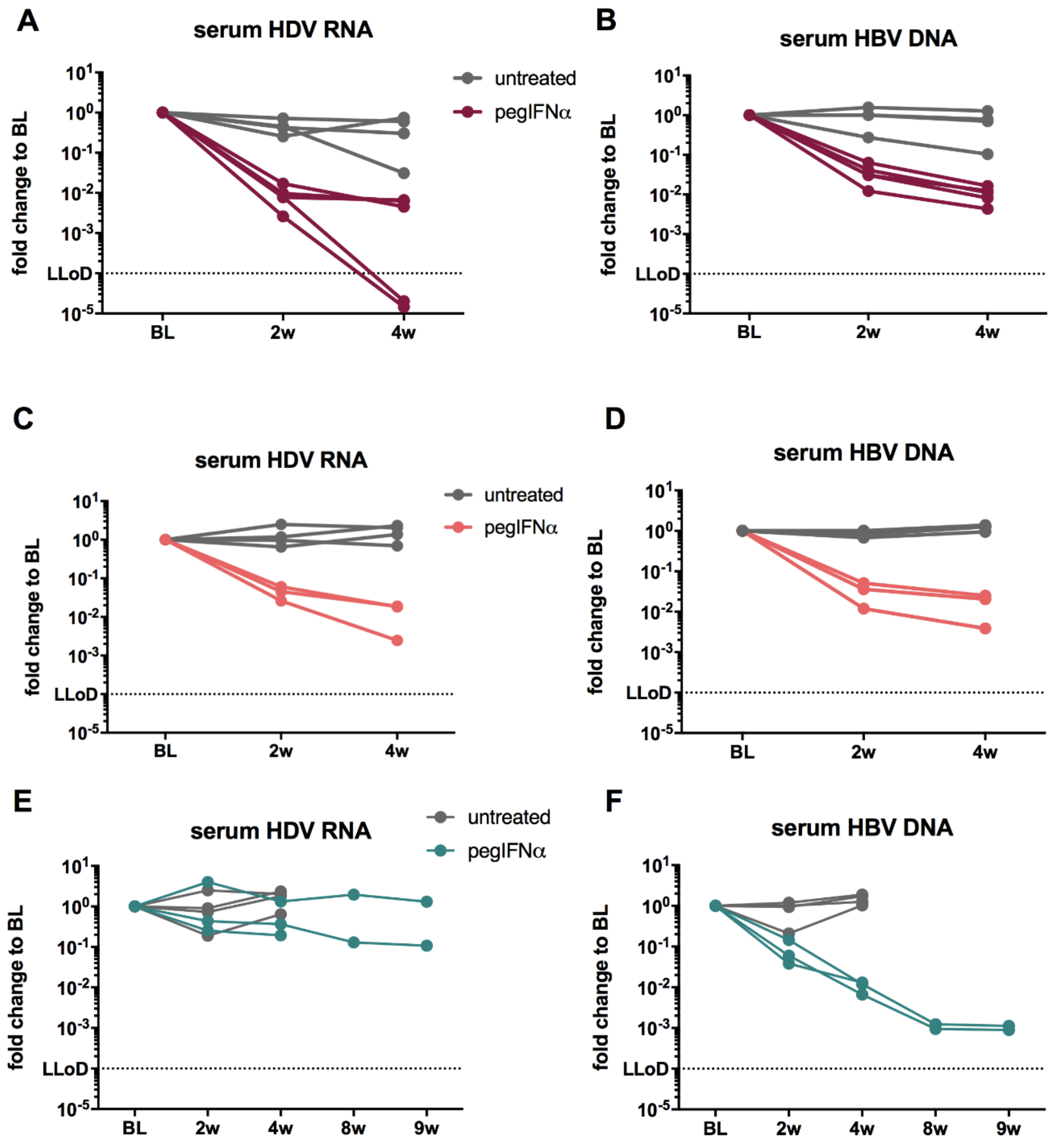

Fig. S1. HDV and HBV viremia in individual mice. Serum HDV RNA (**A, C, E**) and HBV DNA (**B, D, F**) levels depicted as fold change from baseline of individual HBV/HDV-1p- (**A, B**), HBV/HDV-3-infected mice (**C, D**) HBV/HDV-1a- (**E, F**), which were treated with pegIFNα for 4 or 9 weeks or remained untreated.

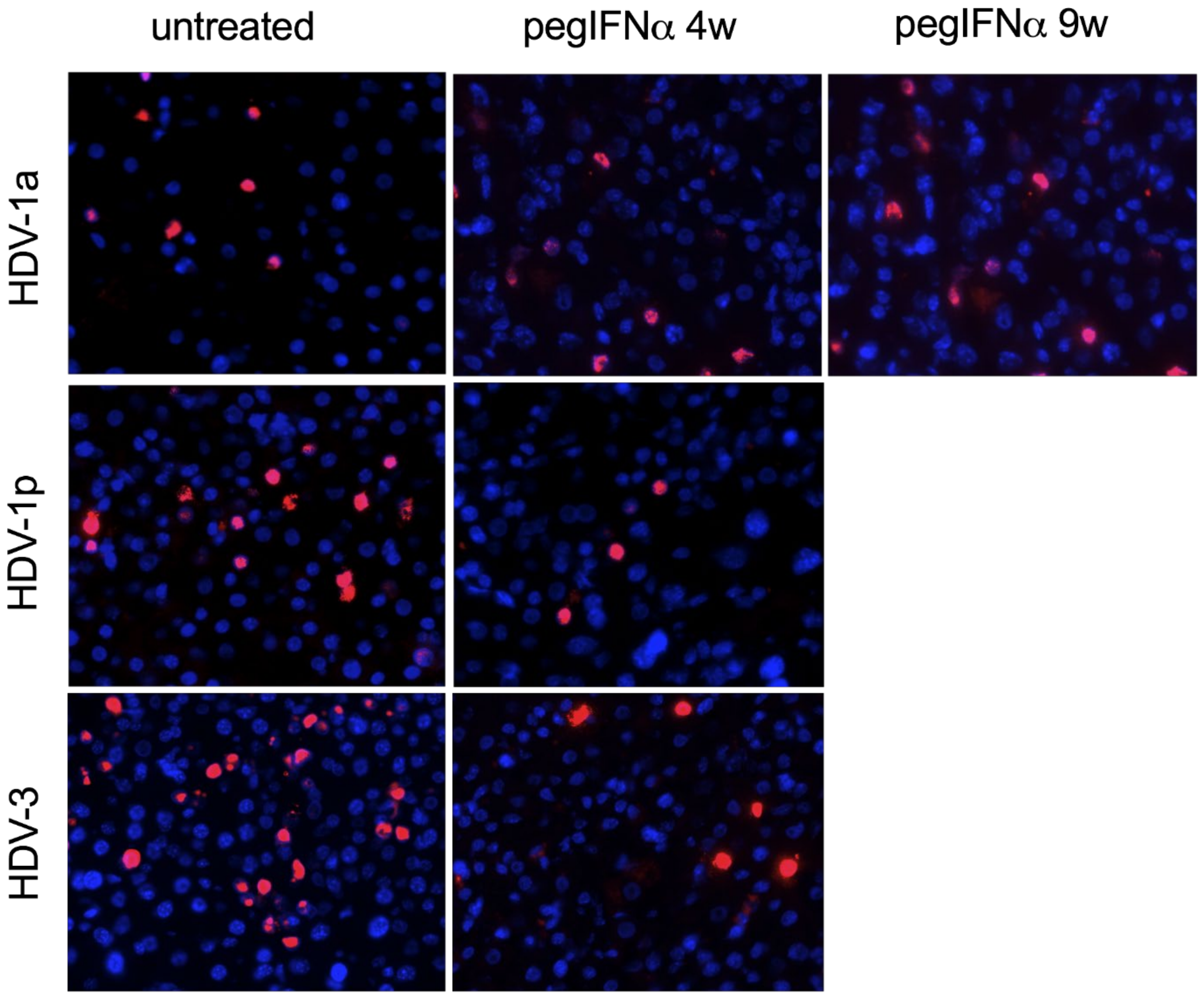

Fig. S2. RNA in situ hybridization in pegIFNα treated mice. B) RNA in situ hybridization of genomic HDV RNA (red) in mouse livers of all groups. Nuclei are stained with Dapi.

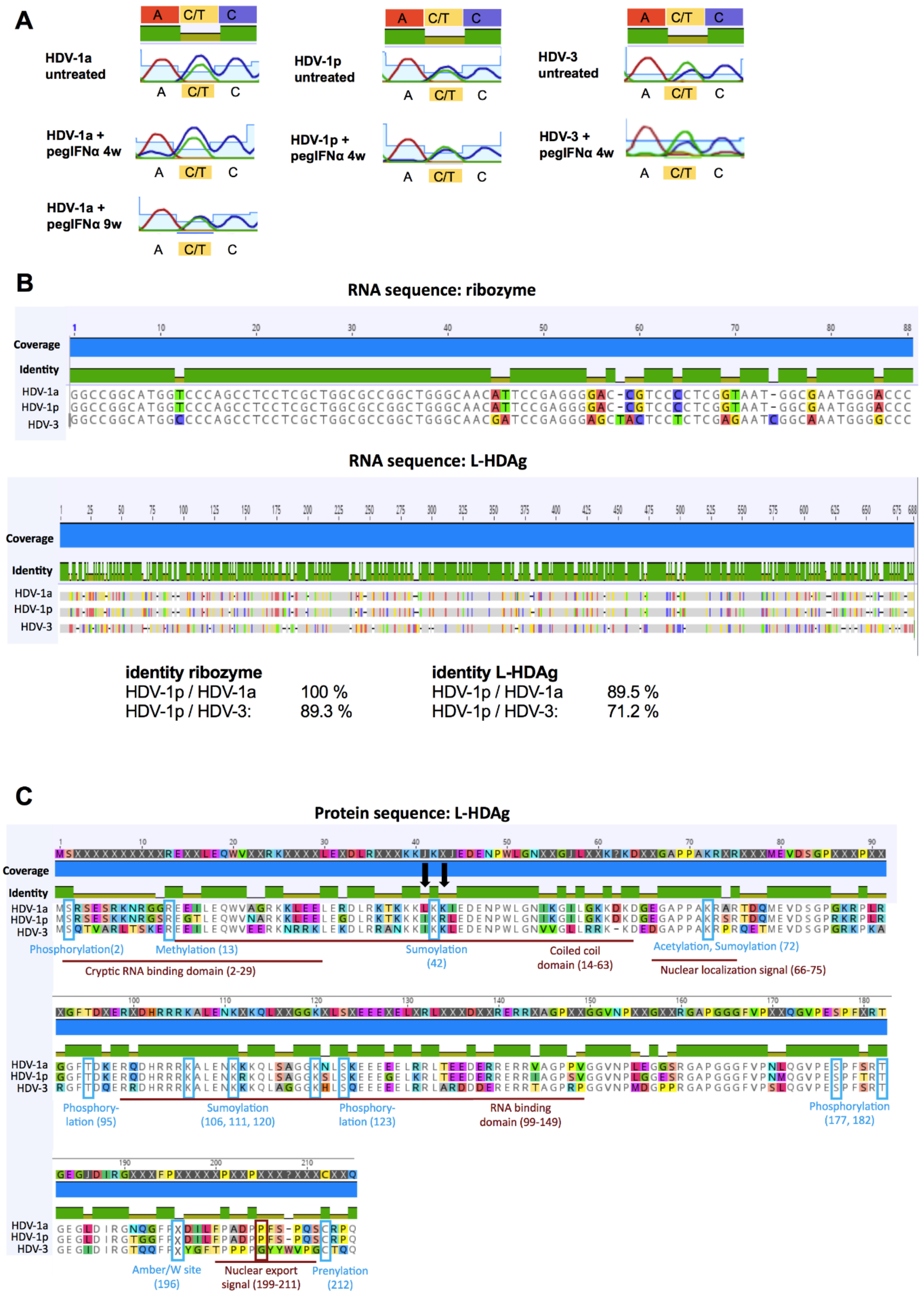

Fig. S3. HDV RNA genome sequencing in HDV-1a, HDV-1p- and HDV-3-infected mice. A) HDV RNA genome sequence of the amber/W site (RNA editing) in untreated and pegIFNα-treated HBV/HDV-1a, HBV/HDV-1p- or HBV/HDV-3-infected mice. ATC (stop codon) encodes

for the S-HDAg, ACC encodes for the L-HDAg. **B)** HDV-1a, HDV-1p and HDV-3 genome sequencing of the open reading frame encoding for the large HDAg (645 nucleotides). **C**) Alignment of the protein sequence of the large HDAg (214 amino acids) of HDV-1a, HDV-1p and HDV-3. Unique differences that exclusively occur in the IFN-resistant HDV-1A-isolate are marked with a black arrow (aa-41, aa-44).

Supplementary references

1. **Lütgehetmann, M.; Mancke**, L.V.; Volz, T.; Helbig, M.; Allweiss, L.; Bornscheuer, T.; Pollok, J.M.; Lohse, A.W.; Petersen, J.; Urban, S.; et al. Humanized Chimeric UPA Mouse Model for the Study of Hepatitis B and D Virus Interactions and Preclinical Drug Evaluation. *Hepatology* **2012**, *55*, 685–694, doi:10.1002/hep.24758.

2. Dandri, M.; Murray, J.M.; Lutgehetmann, M.; Volz, T.; Lohse, A.W.; Petersen, J. Virion Half-Life in Chronic Hepatitis B Infection Is Strongly Correlated with Levels of Viremia. *Hepatology* **2008**, *48*, 1079–1086, doi:10.1002/hep.22469.

3. **Giersch, K.; Bhadra, O.D.**; Volz, T.; Allweiss, L.; Riecken, K.; Fehse, B.; Lohse, A.W.; Petersen, J.; Sureau, C.; Urban, S.; et al. Hepatitis Delta Virus Persists during Liver Regeneration and Is Amplified through Cell Division Both in Vitro and in Vivo. *Gut* **2019**, *68*, 150–157, doi:10.1136/gutjnl-2017-314713.

4. Ferns, R.B.; Nastouli, E.; Garson, J.A. Quantitation of Hepatitis Delta Virus Using a Single-Step Internally Controlled Real-Time RT-QPCR and a Full-Length Genomic RNA Calibration Standard. *J Virol Methods* **2012**, *179*, 189–194, doi:10.1016/j.jviromet.2011.11.001.

5. Pflüger, L.S.; Nörz, D.; Volz, T.; Giersch, K.; Giese, A.; Goldmann, N.; Glebe, D.; Bockmann, J.-H.; Pfefferle, S.; Dandri, M.; et al. Clinical Establishment of a Laboratory Developed Quantitative HDV PCR Assay on the Cobas6800 High-Throughput System. *JHEP Rep* **2021**, *3*, 100356, doi:10.1016/j.jhepr.2021.100356.

6. Malmström, S.; Larsson, S.B.; Hannoun, C.; Lindh, M. Hepatitis B Viral DNA Decline at Loss of HBeAg Is Mainly Explained by Reduced CccDNA Load--down-Regulated Transcription of PgRNA Has Limited Impact. *PLoS One* **2012**, *7*, e36349, doi:10.1371/journal.pone.0036349.

7. Giersch, K.; Homs, M.; Volz, T.; Helbig, M.; Allweiss, L.; Lohse, A.W.; Petersen, J.; Buti, M.; Pollicino, T.; Sureau, C.; et al. Both Interferon Alpha and Lambda Can Reduce All Intrahepatic HDV Infection Markers in HBV/HDV Infected Humanized Mice. *Sci Rep* **2017**, *7*, 3757, doi:10.1038/s41598-017-03946-9.

8. **Giersch, K.; Hermanussen**, L.; Volz, T.; Volmari, A.; Allweiss, L.; Sureau, C.; Casey, J.; Huang, J.; Fischer, N.; Lütgehetmann, M.; et al. Strong Replication Interference Between Hepatitis Delta Viruses in Human Liver Chimeric Mice. *Front Microbiol* **2021**, *12*, 671466, doi:10.3389/fmicb.2021.671466.

9. Pyne, M.T.; Mallory, M.A.; Xie, H.B.; Mei, Y.; Schlaberg, R.; Hillyard, D.R. Sequencing of

the Hepatitis D Virus RNA WHO International Standard. *J Clin Virol* **2017**, *90*, 52–56, doi:10.1016/j.jcv.2017.03.009.

10. Ivaniushina, V.; Radjef, N.; Alexeeva, M.; Gault, E.; Semenov, S.; Salhi, M.; Kiselev, O.; Dény, P. 2001 Hepatitis Delta Virus Genotypes I and II Cocirculate in an Endemic Area of Yakutia, Russia. *Journal of General Virology 82*, 2709–2718, doi:10.1099/0022-1317-82-11-2709.

11. **Giersch, K.; Helbig**, M.; Volz, T.; Allweiss, L.; Mancke, L.V.; Lohse, A.W.; Polywka, S.; Pollok, J.M.; Petersen, J.; Taylor, J.; et al. Persistent Hepatitis D Virus Mono-Infection in Humanized Mice Is Efficiently Converted by Hepatitis B Virus to a Productive Co-Infection. *J Hepatol* **2014**, *60*, 538–544, doi:10.1016/j.jhep.2013.11.010.

12. **Allweiss, L.; Gass**, S.; Giersch, K.; Groth, A.; Kah, J.; Volz, T.; Rapp, G.; Schöbel, A.; Lohse, A.W.; Polywka, S.; et al. Human Liver Chimeric Mice as a New Model of Chronic Hepatitis E Virus Infection and Preclinical Drug Evaluation. *J Hepatol* **2016**, *64*, 1033–1040, doi:10.1016/j.jhep.2016.01.011.
