# Supplementary material for: Strain-specific responsiveness of hepatitis D virus to interferon-alpha treatment
Source: JHEP Rep. 2023 Jan 24;5(4):100673. doi: 10.1016/j.jhepr.2023.100673 (PMC9996322; doi:10.1016/j.jhepr.2023.100673)
Supplement: Multimedia component 2 [file mmc2.docx]

**JHEP Reports**

**CTAT methods**

Tables for a “Complete, Transparent, Accurate and Timely account” (CTAT) are now mandatory for all revised submissions. The aim is to enhance the reproducibility of methods.

- Only include the parts relevant to your study
- Refer to the CTAT in the main text as ‘Supplementary CTAT Table’
- Do not add subheadings
- Add as many rows as needed to include all information
- Only include one item per row

**If the CTAT form is not relevant to your study, please outline the reasons why:**

|  |
| --- |

- 1. **Antibodies**

| **Name** | **Citation** | **Supplier** | **Cat no.** | **Clone no.** |
| --- | --- | --- | --- | --- |
| rabbit anti-Delta antibody |  | John Taylor | N/A |  |
| human anti-Delta antibody |  | Patient | N/A |  |
| rabbit anti-HBcAg |  | Dako | B0586 |  |
| mouse anti-human-albumin antibody |  | Sigma-Aldrich | A6684 |  |
| Dako Envision+ system HRP-labeled polymer anti-rabbit |  | Dako | K4003 |  |
| Mouse anti-CK18 |  | Dako | sc-6259 | DC10 |

- 1. **Cell lines**

| **Name** | **Citation** | **Supplier** | **Cat no.** | **Passage no.** | **Authentication test method** |
| --- | --- | --- | --- | --- | --- |
| HepG2^hNTCP^ cells | Drexler JF, Geipel A, Konig A, Corman VM, van Riel D, Leijten LM, et al. Bats carry pathogenic hepadnaviruses antigenically related to hepatitis B virus and capable of infecting human hepatocytes. Proc Natl Acad Sci U S A 2013;110:16151-16156**.** |  |  | unknown | NBD-Taurocholate uptake assay; PCR |

- 1. **Organisms**

| **Name** | **Citation** | **Supplier** | **Strain** | **Sex** | **Age** | **Overall n number** |
| --- | --- | --- | --- | --- | --- | --- |
| urokinase-type plasminogen activator (uPA)/severe combined immunodeficiency (SCID)/beige/interleukin-2 receptor gamma chain negative (IL2RG^–/–^) mice | Dandri et al., Hepatology. 2001 doi: 10.1053/jhep.2001.23314 | II2rg ko mice (JAX Mice stock number 003169; C.129S4-Il2rg<tmlWjl>/J) were 10 times backcrossed on uPA/SCID mice, which originate from crossing uPA mice (Jax Stock JR002214 (not available anymore); B6SJL-TgN(Alb1Plau)144Bri) for 10 generations on SCID beige mice (Taconic model: CBSCBG; C.B-Igh-1b/GbmsTac-Prkdcscid-Lystbg N7) |  | **m/f** | **14-20 weeks** | **40** |

- 1. **Sequence based reagents**

| **Name** | **Sequence** | **Supplier** |
| --- | --- | --- |
| HDV_F | GCGCCGGCYGGGCAAC | IDT DNA |
| HDV_R | TTCCTCTTCGGGTCGGCATG | IDT DNA |
| HDV_Probe | FAM-CGCGGTCCGACCTGGGCATCCG-BHQ | IDT DNA |
| HDV_F_bio | biotin-GCGCCGGCYGGGCAAC | IDT DNA |
| HDV_R_bio | biotin-TTCCTCTTCGGGTCGGCATG | IDT DNA |
| HBV (S) | Pa03453406_s1 | Thermo Fischer Scientific |
| h- beta-globin | Hs00758889_s1 | Thermo Fischer Scientific |
| hGAPDH | Hs99999905_m1 | Thermo Fischer Scientific |
| hRLP30 | Hs00265497_m1 | Thermo Fischer Scientific |
| hISG15 | Hs00192713_m1 | Thermo Fischer Scientific |
| hISG20 | Hs00158122_m1 | Thermo Fischer Scientific |
| hOAS1 | Hs00973637_m1 | Thermo Fischer Scientific |
| hMxA | Hs00895608_m1 | Thermo Fischer Scientific |
| hHLA-E | Hs03045171_m1 | Thermo Fischer Scientific |
| hADAR | Hs01017595_g1 | Thermo Fischer Scientific |
| hCXCL10 | Hs00171042_m1 | Thermo Fischer Scientific |
| hTGF-β | Hs00171257_m1 | Thermo Fischer Scientific |
| hIL28AB | Hs04193049_gH | Thermo Fischer Scientific |
| hSTAT1 | Hs01013989_m1 | Thermo Fischer Scientific |
| hMDA5 | Hs01070332_m1 | Thermo Fischer Scientific |
| hCasp8 | Hs01018151_m1 | Thermo Fischer Scientific |
| hNTCP | Hs00914889_m1 | Thermo Fischer Scientific |
| hISG15 | Hs00192713_m1 | Thermo Fischer Scientific |
| hISG20 | Hs00158122_m1 | Thermo Fischer Scientific |
| ISH probe HDV | ACD assay number: 484611 | Advanced Cell Diagnostics, Inc |
| ISH probe -1AJ genomic (G) HDV RNA | ADC assay number: 478131 | Advanced Cell Diagnostics, Inc |
| ISH probe HDV-1AJ antigenomic (AG) HDV RNA | ADC assay number :475311 | Advanced Cell Diagnostics, Inc |

- 1. **Biological samples**

| **Description** | **Source** | **Identifier** |
| --- | --- | --- |
| HDV-1a (GT 1) | Cell culture virus | Hamburg (M21012) |
| HDV-1p (GT 1) | Cell culture virus | Hamburg (OL825606) |
| HDV-3 (GT 3) | Cell culture virus | Hamburg (L22063) |

- 1. **Deposited data**

| **Name of repository** | **Identifier** | **Link** |
| --- | --- | --- |
|  |  |  |

- 1. **Software**

| **Software name** | **Manufacturer** | **Version** |
| --- | --- | --- |
| BioRender | BioRender |  |
| GraphPad Prism | Graphpad Software | Version 9.3 |

- 1. **Other (*e.g*. drugs, proteins, vectors etc.)**

| pegIFNα (Pegasys) | Roche, Basel, Switzerland |  |
| --- | --- | --- |
|  |  |  |

- 1. **Please provide the details of the corresponding methods author for the manuscript:**

| Katja Giersch: [kgiersch@uke.de](mailto:kgiersch@uke.de) |
| --- |

**2.0 Please confirm for randomised controlled trials all versions of the clinical protocol are included in the submission. These will be published online as supplementary information.**

|  |
| --- |
