# Supplementary material for: Strain-specific responsiveness of hepatitis D virus to interferon-alpha treatment
Source: JHEP Rep. 2023 Jan 24;5(4):100673. doi: 10.1016/j.jhepr.2023.100673 (PMC9996322; doi:10.1016/j.jhepr.2023.100673)
Supplement: Multimedia component 4 [file mmc4.pdf]

Research article JHEP|Reports

Strain-specific responsiveness of hepatitis D virus to interferon-alpha treatment

Katja Giersch,[1] Paulina Perez-Gonzalez,[1] Lennart Hendricks,[1] Nora Goldmann,[2] Jonathan Kolbe,[1] Lennart Hermanussen,[1] Jan-Hendrick Bockmann,[1,3] Tassilo Volz,[1] Annika Volmari,[1] Lena Allweiss,[1,3] Joerg Petersen,[4] Dieter Glebe,[2,3] Marc Lütgehetmann,[3,5,†] Maura Dandri[1,3,*,†]

[1]Department of Internal Medicine, University Medical Center Hamburg-Eppendorf, Hamburg, Germany; [2]Institute of Medical Virology, National Reference Center for Hepatitis B Viruses and Hepatitis D Viruses, Justus Liebig University Giessen, Giessen, Germany; [3]Hamburg-Lübeck-Borstel-Riems and Giessen-Marburg-Langen Partner Sites, Germany; [4]IFI Institute for Interdisciplinary Medicine at Asklepios Clinic St. Georg, Hamburg, Germany; [5]Department of Medical Microbiology, Virology and Hygiene, University Medical Center Hamburg-Eppendorf, Hamburg, Germany

JHEP Reports **2023.** https://doi.org/10.1016/j.jhepr.2023.100673

Background & Aims: Pegylated interferon alpha (pegIFNα) is commonly used for the treatment of people infected with HDV. However, its mode of action in HDV-infected cells remains elusive and only a minority of people respond to pegIFNα therapy. Herein, we aimed to assess the responsiveness of three different cloned HDV strains to pegIFNα. We used a previously cloned HDV genotype 1 strain (dubbed HDV-1a) that appeared insensitive to interferon-α *in vitro*, a new HDV strain (HDV-1p) we isolated from an individual achieving later sustained response to IFNα therapy, and one phylogenetically distant genotype 3 strain (HDV-3).
Methods: PegIFNα was administered to human liver chimeric mice infected with HBV and the different HDV strains or to HBV/HDV infected human hepatocytes isolated from chimeric mice. Virological parameters and host responses were analysed by qPCR, sequencing, immunoblotting, RNA *in situ* hybridisation and immunofluorescence staining.
Results: PegIFNα treatment efficiently reduced HDV RNA viraemia (~2-log) and intrahepatic HDV markers both in mice infected with HBV/HDV-1p and HBV/HDV-3. In contrast, HDV parameters remained unaffected by pegIFNα treatment both in mice (up to 9 weeks) and in isolated cells infected with HBV/HDV-1a. Notably, HBV viraemia was efficiently lowered (~2-log) and human interferon-stimulated genes similarly induced in all three HBV/HDV-infected mouse groups receiving pegIFNα. Genome sequencing revealed highly conserved ribozyme and L-hepatitis D antigen post-translational modification sites among all three isolates.
Conclusions: Our comparative study indicates the ability of pegIFNα to lower HDV loads in stably infected human hepatocytes *in vivo* and the existence of complex virus-specific determinants of IFNα responsiveness.
Impact and implications: Understanding factors counteracting HDV infections is paramount to develop curative therapies. We compared the responsiveness of three different cloned HDV strains to pegylated interferon alpha in chronically infected mice. The different responsiveness of these HDV isolates to treatment highlights a previously underestimated heterogeneity among HDV strains.
© 2023 The Author(s). Published by Elsevier B.V. on behalf of European Association for the Study of the Liver (EASL). This is an open access article under the CC BY license (http://creativecommons.org/licenses/by/4.0/).

Introduction

The hepatitis delta virus (HDV) infects around 20 million people worldwide[1] and recent reports suggested that the number of HDV-positive individuals may be even higher.[1–3] Liver disease associated with chronic hepatitis D (CHD) causes substantial global morbidity (cirrhosis, hepatocellular carcinoma) and mortality.[4] HDV RNA replication takes place in the nucleus of hepatocytes by hijacking the host RNA polymerase II, which amplifies the genomic HDV RNA through a double rolling-circle amplification process.[5] During HDV replication, two additional viral RNAs accumulate in the hepatocytes: the antigenomic RNA, which is an exact complement of the genomic RNA and the smaller linear mRNA encoding for the hepatitis delta antigen (HDAg). HDAg binds specifically to the HDV RNA and exists in two different forms: the small HDAg (S-HDAg) that is important for virus replication and the large variant (L-HDAg), which can inhibit replication and promotes virus assembly through a prenylation site.[5] The L-HDAg is generated by post-transcriptional RNA editing at the adenosine 1012 (amber/W site), which is mediated by the RNA-specific adenosine deaminase (ADAR). The balance between viral replication and assembly is orchestrated by the presence of S- and L-HDAg and by post-translational modifications of these proteins, such as prenylation, phosphorylation, methylation, and SUMOylation.[6] HDV is a satellite virus that requires expression of hepatitis B virus (HBV) envelope

Keywords: HDV; Human liver chimeric mice; Resistance; Antiviral; Genotype.
Received 29 June 2022; received in revised form 2 December 2022; accepted 3 January 2023; available online 24 January 2023
† These authors contributed equally to this work.
* Corresponding author. Address: I. Dept. of Internal Medicine, Center for Internal Medicine, University Medical Center Hamburg-Eppendorf, Martinistrasse 52, D 20246 Hamburg, Germany. Tel.: +49-40–7410-52949; fax: +49-40–7410-57232.
E-mail address: m.dandri@uke.de (M. Dandri).

ELSEVIER
EASL The Home of Hepatology

proteins to release infectious HDV particles.[7] Both HBV and HDV infect the human hepatocytes via the sodium taurocholate co-transporting polypeptide (NTCP).[8] Moreover, and in strong contrast to HBV, HDV can also disseminate through cell division.[9,10] Active HDV infection either can occur upon simultaneous co-infection with HBV or as a super-infection in people already infected with HBV. HDV is classified into eight genotypes. Although HDV-1 is the most prevalent genotype worldwide, HDV-3 is frequently associated with the most severe hepatitis.[11,12] Sequence divergence among the genotypes is as high as 40% over the entire RNA genome with the greatest difference observed between HDV genotype 1 (HDV-1) and genotype 3 (HDV-3).[13]

Because of its compact genomic organisation and lack of its own polymerase, HDV offers very few therapeutic targets and current HBV therapies based on the use of nucleos(t)ide analogues (NUCs) inhibiting the HBV polymerase cannot directly target HDV. Understanding the factors that are able to block HDV infection and replication is therefore of utmost importance for the development of HDV curative therapies. In 2020, the HBV/HDV entry inhibitor bulevirtide (Hepcludex/Myrcludex-B) obtained conditional marketing authorisation by the EMA as the first HDV-specific drug,[14] and pegylated interferon lambda and the prenylation inhibitor lonafarnib are currently being tested in clinical trials.[15] Aside such advances, pegylated interferon-alpha (pegIFNα) has been the most commonly used off-label treatment against HDV for decades. However, treatment is associated with substantial side effects and leads to sustained virological response (defined as undetectable serum HDV RNA 6 months after treatment) in only about 25–30% of patients, with high relapse rates after treatment cessation. Nevertheless, various clinical trials are currently evaluating the contribution of IFN treatment to HDV therapy in novel combination regimens.[16] Apart from acting as an immunomodulatory agent, IFNα induces interferon stimulated genes (ISGs) via the janus kinase-signal transducer and activator of transcription (JAK-STAT) signalling pathway also in hepatocytes, thereby triggering a cellular antiviral state.[17] Nonetheless, the mode of action (MoA) of IFNα in stably HDV-infected primary hepatocytes remains elusive. HDV is sensed by the pattern recognition receptor melanoma differentiation antigen 5 (MDA5) of the hepatocytes[18] leading to ISG enhancement and chemokine production in HBV/HDV infected cells.[19,20] However, such antiviral state does not limit HDV replication, whereas therapeutically applied more stable (pegylated) IFNs could affect a patient-derived HDV inoculum *in vivo*.[17] Moreover, IFNα was shown to promote silencing of the HBV genome, thus lowering HBV transcript levels[21] and to destabilise HDV RNA during cell division.[22]

To date, only a limited amount of HDV strains have been cloned[23–27] Most studies used a peculiar HDV-1 clone of uncertain human origin[28,29] here dubbed HDV-1a (Table 1 and methods), which turned out to be unaffected by IFNα *in vitro*,[18,30,31] leading to the assumption that interferon only marginally impairs HDV RNA replication in stably infected cells.[22] In this study, we assessed and compared the antiviral and intrinsic host response to pegIFNα treatment *in vivo* using human liver chimeric mice stably infected with HBV and either with HDV-1a or two different human-derived cloned HDV strains: a novel genotype 1 (HDV-1p) and an HDV-3 (Table 1).[32]

Participants and methods

Virus generation

The patient-derived HDV-1 isolate (HDV-1p) was isolated from a male individual with CHD from the university clinic of Hamburg,[33] passaged in human liver chimeric uPA/SCID/beige/IL2RG$^{-/-}$ (USG) mice, sequenced, cloned as genome-sense tandem dimer in pcDNA3.1(+), and infectious particles were produced in cell culture (Table 1). The HDV-3 isolate (Peru-1) was obtained from a young man from Peru, who developed severe acute hepatitis, which was cloned by Casey *et al.*[32] The origin of the first HDV clone available in the research community is less clear: it is a genotype 1 strain, individual(s) sera were passaged through various chimpanzees at NIH (J. Taylor, pers. commun.), inoculated in a woodchuck and then cloned (Table 1). For the production of the HDV-1a and HDV-3 strains, the HDV recombinant plasmid pSVL(D3) (kindly provided by J. Taylor, Philadelphia, PA, USA)[28] and pCMV3-Peru-1.2 (kindly provided by J. Casey, Washington DC, USA)[34]

Table 1. Characteristics of the source individuals and cloned viruses.

	HDV-1a	HDV-1p	HDV-3peru
Sex	Male	Male	Male
Age	Unknown	52 years	18 years
Origin	Unclear	Turkey	Peru
Clinical characteristics	Unclear	Chronic HBV and HDV infection	Severe acute HBV and HDV infection
IFNα response	Untreated	Responsive	Untreated
Specifics	Serially passaged in chimpanzees, in a woodchuck, and then cloned	NA treatment when serum was collected; serially passaged in HBV-infected humanised mice and then cloned	Not passaged; initially HBV envelope of genotype F
Accession number	M21012 [29]; AJ00058 [38][a]	OL825606	L22063
Year virus cloning	1988	2019	1993
HDV plasmid for cloning	HDV trimer in pSVL	HDV tandem dimer in pcDNA3.1(+)	pCMV3-Peru-1.2
HBV envelope-expressing plasmid for cloning	pT7HB2.7 (genotype D)	HBV subgenome in pcDNA3.1(+) (genotype D)	pT7HB2.7 (genotype D)
References	[29,38]	[17,33] and this study	[34]

Summary of information available regarding the three HDV strains used in this study, including known participant characteristics, cloning strategies used, referring accession numbers, and references.
aSequence analyses revealed that this HDV-1 clone shows 99.7% identity with M21012 and 99.5% with AJ00058. IFN, interferon; NA, nucleos(t)ide analogue.

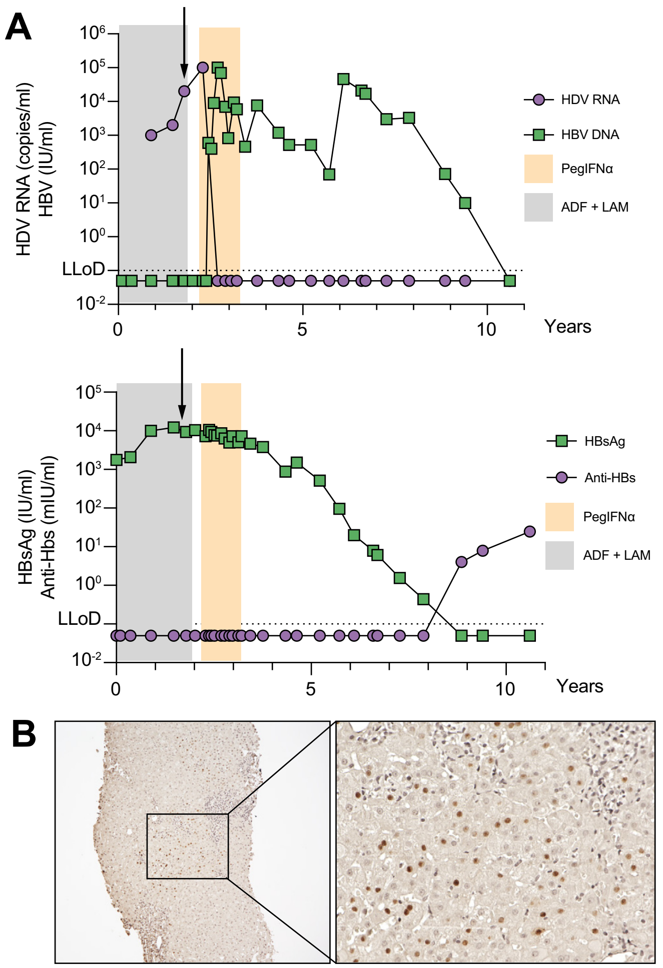

Fig. 1. Participant characteristics (HDV-1p). (A) HDV and HBV viraemia (above) and HBsAg and anti-HBs levels (below) during and after NUC (grey box) and pegIFNα treatment (orange box) of the infected individual. The black arrow indicates the time point when serum was collected to infect human liver chimeric mice for passaging the virus. (B) Immunohistology of HDAg (brown nuclei) in a liver biopsy of the individual before treatment with pegIFNα (overview, 10× and close up, 20×). ADF, adefovir; anti-HBs, hepatitis B surface antibody; hAAT, human alpha antitrypsin; HDAg, hepatitis D antigen; HBsAg, hepatitis B virus surface antigen; LAM, lamivudine; pegIFNα, pegylated interferon alpha.

were used. Infectious HDV-1a, HDV-1p, and HDV-3 particles were generated in HuH7 cells as previously described.[35] In brief, cells were transfected with equimolar amounts of HDV-1a, HDV-1p, or HDV-3 recombinant plasmids and the HBV envelope-expressing vectors pcDNA3.1(+)[36] (HDV-1p) or pT7HB2.7[37] (HDV-1a, HDV-3) encoding the surface proteins of HBV genotype D using Fugene HD Transfection Reagent (Promega, Madison, WI, USA) (Table 1).

Treatment

In vivo, pegIFNα treatment was started when HBV/HDV-1a-, HBV/HDV-1p-, or HBV/HDV-3-infected mice reached stable HBV and HDV viraemia levels. Mice received pegIFNα (Pegasys; Roche, Basel, Switzerland) twice a week subcutaneously (25 ng/g body weight)[17,21] for 4 or 9 weeks. Mice were sacrificed 24 h after the last pegIFNα injection. Cultured primary human hepatocytes (PHHs) (isolated from an HBV/HDV-infected mouse) received IFNα (1,000 IU/ml; Roferon-A; Roche, Basel, Switzerland) starting 1 day after plating. The culture medium was changed twice a week.

More methods (*e.g.* generation and infection of mice, virological measurements, RNA *in situ* hybridisation) can be found in the supplementary information.

Results

Production and infectivity of HDV-1p *in vitro* and *in vivo*

As summarised in Table 1, the HDV-1p strain was obtained from a person with CHD receiving nucleos(t)ide analogues (NUCs) treatment (lamivudine plus adefovir), which resulted in undetectable serum HBV DNA levels (Fig. 1A). HDV viraemia was detectable and immunohistology confirmed an abundance of HDAg-positive hepatocytes (Fig. 1A and C). Eventually, after discontinuation of NUC treatment, the individual received pegIFNα for 48 weeks, which resulted in a sustained HDV response and temporary increase of HBV viraemia,[33] but led to HBV and HBsAg loss and seroconversion to hepatitis B surface antibody years later (Fig. 1B).

The HDV-positive isolate obtained before IFN treatment was first shown to be infectious in HBV-infected human liver chimeric USG mice[17] and now sequenced and cloned as described in the Participants and methods section (Table 1). The full genome sequence of the HDV-1p strain is available at NCBI (accession number: OL825606). HDV-1p virus stocks were produced in HuH7 cells and their infectivity was tested first *in vitro* using HepG2^{hNTCP} cells. Seven days after HDV-1p inoculation (multiplicity of infection; MOI = 1), HDAg staining (Fig. 2A) confirmed *in vitro* infectivity of the HDV-1p particles produced after cloning the virus. The *in vitro* infectivity of HDV-1p appeared similar to the cloned strains HDV-3 (MOI = 1) and HDV-1a (MOI = 2) (Fig. 2A).

To assess the infectivity of the cloned HDV-1p strain *in vivo*, HBV-infected humanised USG mice were super-infected with HDV-1p from the supernatant of HuH7 cells (4×10^6 GE/mouse). In these HBV infected mice, where HDV infection is first established in a low number of PHHs, HDV disseminated among PHHs and viraemia increased up to week 6 after super-infection and remained stable until the end of the observation time (9 weeks post super-infection) (Fig. 2B). In line with previous studies[39,40] we observed a decrease of HBV viraemia (fivefold) during HDV-1p super-infection (data not shown), while HDAg was clearly detected in human hepatocytes (Fig. 2C). Moreover, amounts and distribution of S- and L-HDAg resembled those detected in the patient liver biopsy (Fig. 2D). Likewise, HDV-3 and HDV-1a reached stable HDV titres after around 5 weeks of HDV super-infection and showed similar ratios of S- and L-HDAg (Fig. 2B and D). The amount of HDV-infected human hepatocytes determined by immunofluorescence tended to be higher in mice infected with HDV-3 (63%) compared with those infected with HDV-1a (32%) or HDV-1p (42%), suggesting that HDV-3 may display superior spreading capacities *in vivo* than the HDV-1 strains. Overall, these comparative analyses show that human liver chimeric mice are well suited to study patient-derived viral strains.

PegIFNα treatment in mice infected with different HDV strains

Human liver chimeric mice stably infected with HBV and one of the three HDV clones received pegIFNα twice a week (Fig. 3A). In

HBV/HDV-1p and HBV/HDV-3 infected mice, 4-week pegIFNα treatment reduced HDV viraemia by more than 2.0-log and by 1.7-log, HBV viraemia by 2.0-log and 1.7-log, respectively, as well as levels of circulating HBsAg (Fig. 3B–D, and Fig. S1A–D). Intrahepatic analyses revealed a clear reduction of HDV RNA (HDV-1p: 0.9-log, HDV-3: 1.5-log) (Fig. 3E) and HBV pgRNA levels relative to human hepatocyte contents (Fig. 3F) in mice infected with HBV/HDV-1p (0.5-log) or HBV/HDV-3 (0.6-log) in comparison with untreated control mice. In striking contrast, 4-week treatment of HBV/HDV-1a infected mice clearly reduced HBV DNA (2-log) and HBsAg in serum (Fig. 3C and D and Fig. S1B); intrahepatic pgRNA (1.5-log) (Fig. 3E), but had no effect

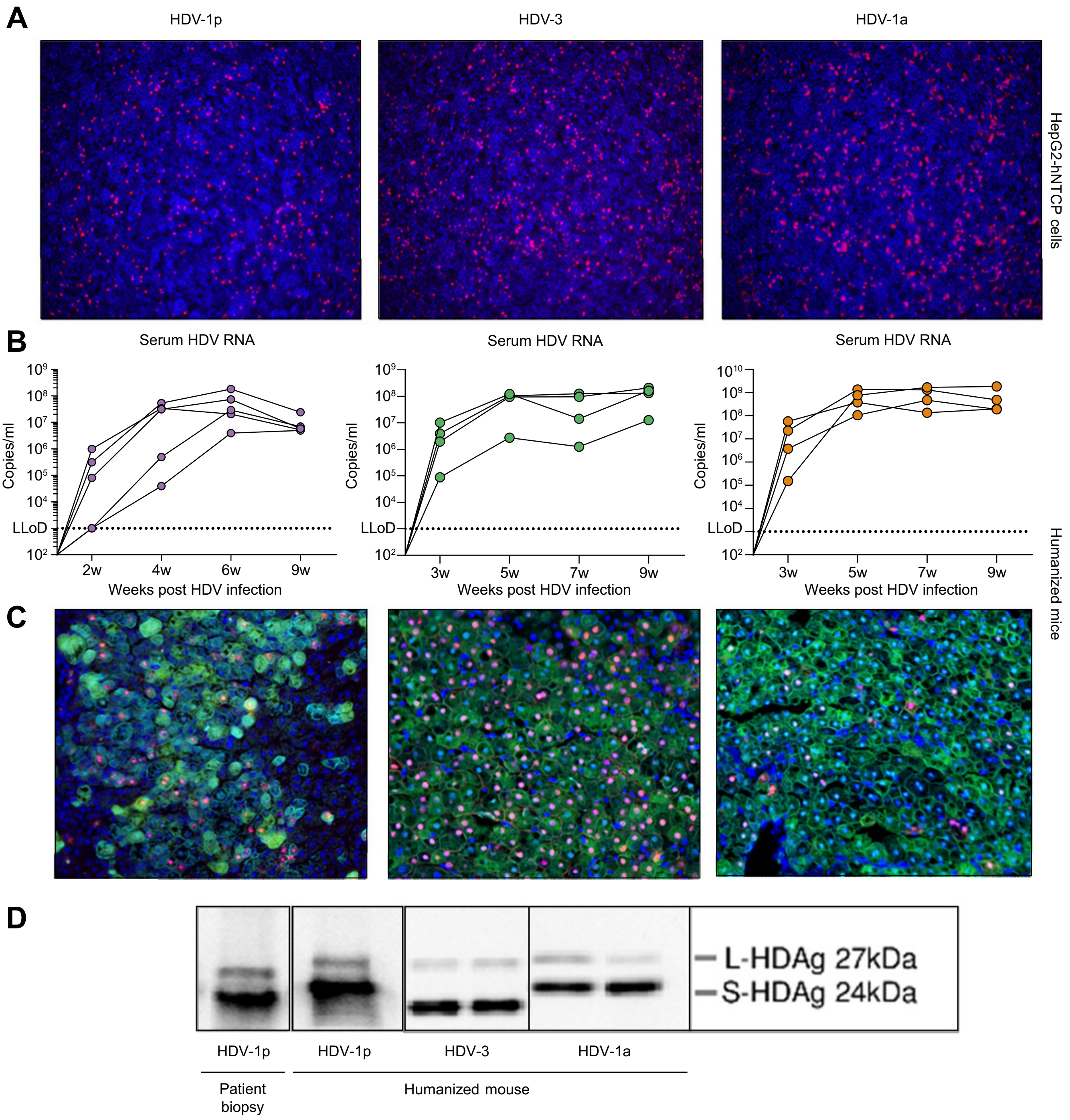

Fig. 2. *In vitro* and *in vivo* infectivity of the cloned strains HDV-1p, HDV-3, and HDV-1a. (A) HDAg (red, immunofluorescence [IF] staining) in HepG2^{hNTCP} cells 7 days after mono-infection with HDV-1p, HDV-3, or HDV-1a. (B) Development of HDV viraemia in humanised mice (HDV-1p left, HDV-3 middle, HDV-1a right). IF staining of HDAg (red), HBcAg (green), and the human marker CK18 (turquoise) (C) and Western blot analysis of S- and L-HDAg (D) in stable HBV/HDV-infected mice and in the liver biopsy obtained from the individuals with HDV-1p infection. HDAg, hepatitis D antigen.

on HDV in serum (Fig. 3B and Fig. S1E) and liver (Fig. 3E). Even after extending pegIFNα treatment to 9 weeks, serum HDV RNA levels remained comparable in treated and untreated mice harbouring the HDV-1a strain (Fig. 3B and Fig. S1E), while HBV viraemia (3-log) and HBsAg levels decreased even further (Fig. 3C and D, Fig. S1F).

In line with liver HDV RNA levels, immunofluorescence staining of HDV-1p- and HDV-3-infected mice 4 weeks after pegIFNα

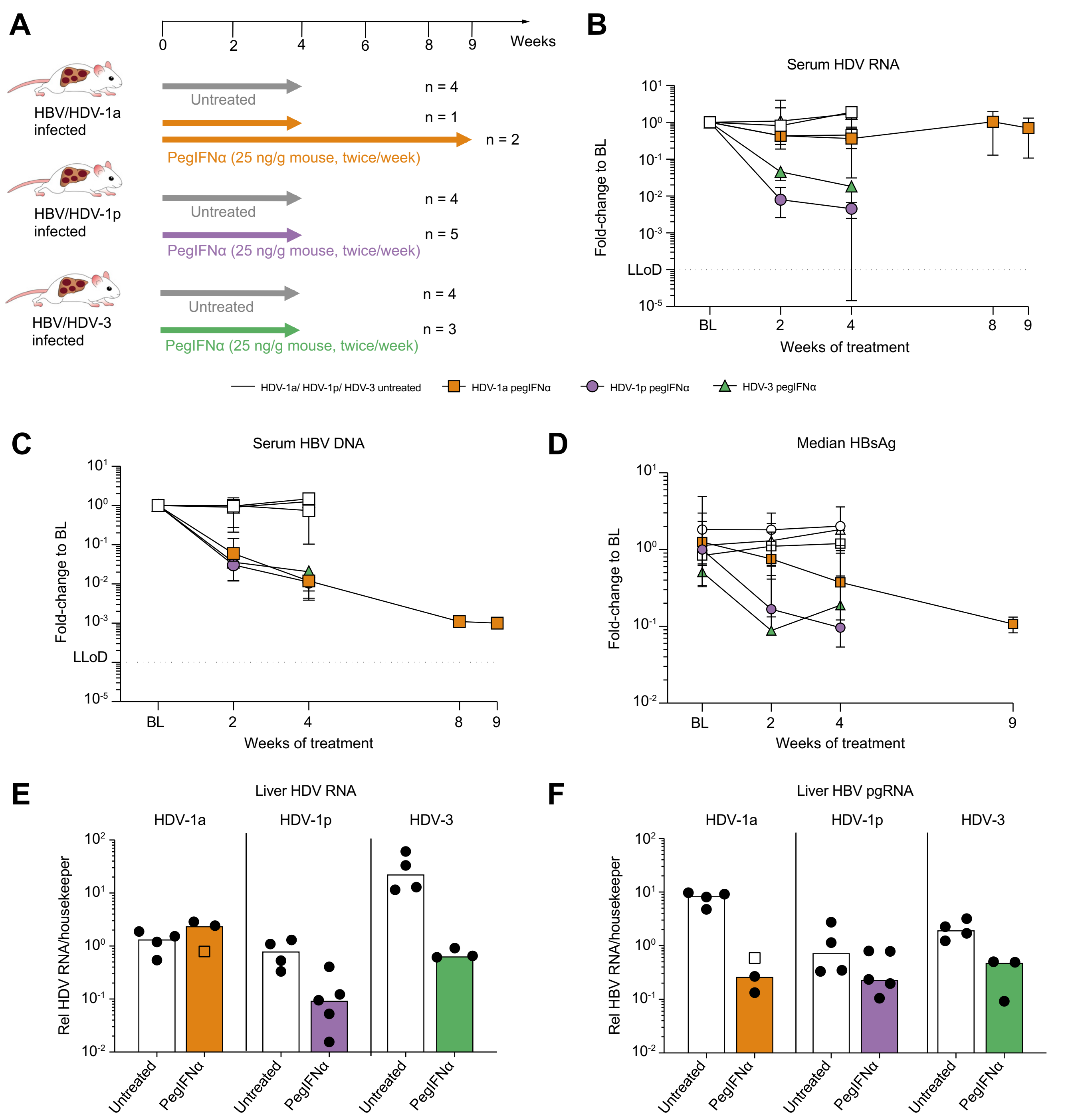

Fig. 3. PegIFNα treatment in HBV/HDV-1a, HBV/HDV-1p, and HBV/HDV-3 infected mice. (A) Experimental design: stable HBV/HDV-1a-, HBV/HDV-1p-, and HBV/HDV-3-infected mice received pegIFNα for 4 weeks or remained untreated. Some mice infected with HBV/HDV-1a were treated for 9 weeks. Serum HDV (B), HBV (C), and HBsAg (D) are depicted as fold change from baseline in HBV/HDV-1a-infected untreated (grey line, square symbols; n = 4) or treated mice (blue line, square symbols; n = 3), in HBV/HDV-1p-infected untreated (grey line, round symbols; n = 4) or treated mice (pink line, round symbols; n = 5) and in HBV/HDV-3-infected untreated (grey line, triangular symbols; n = 4) or treated mice (light red line, triangular symbols; n = 5). Curves show median levels with range. PCR measurements of liver HDV RNA (E) and HBV pgRNA (F) in untreated and treated HBV/HDV-1a, HBV/HDV-1p, and HBV/HDV-3 infected mice. Bars show median levels black dots represent individual mice. HBV/HDV-1a infected mice are shown as clear square or black dots when they were treated for 4 (n = 1) or 9 weeks (n = 2), respectively. BL, baseline; HBsAg, hepatitis B virus surface antigen; LLoD, lower limit of detection; pegIFNα, pegylated interferon alpha.

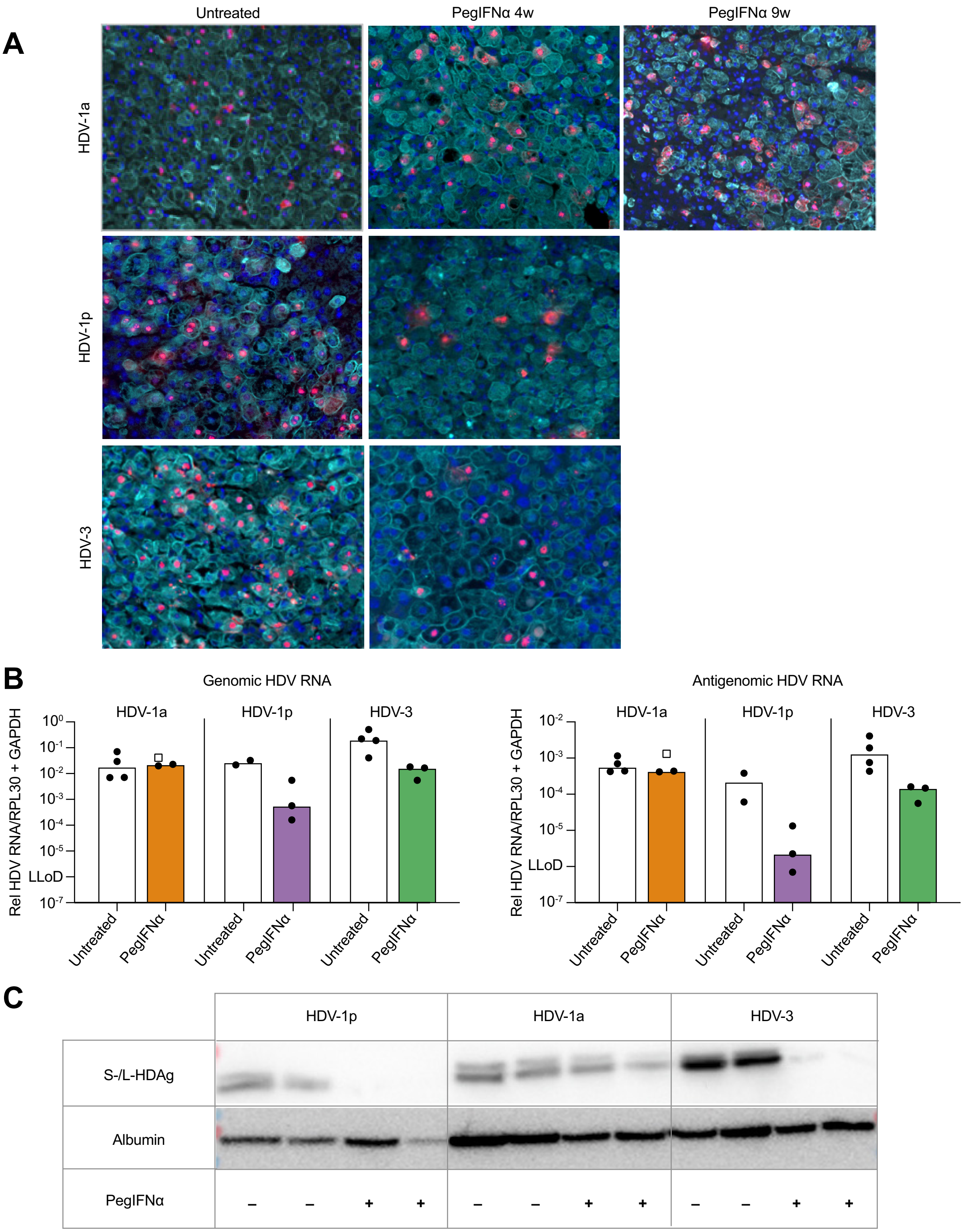

Fig. 4. Immunofluorescence staining, genomic/antigenomic HDV RNA and S-/L-HDAg. (A) Immunofluorescence staining of HDAg (red) and CK18 (human hepatocytes, aqua) in mouse livers of all groups. Nuclei are stained with Hoechst 33258 (blue). (B) Genomic (left) and antigenomic HDV RNA (right) (qPCR assay using biotinylated magnetic beads) in untreated and treated mice infected with HBV/HDV-1a, HBV/HDV-1p, and HBV/HDV-3. HBV/HDV-1a-infected mice are shown as clear square or black dots when they were treated for 4 (n = 1) or 9 weeks (n = 2), respectively. (C) Western blot analysis of S-HDAg (23 kDa) and L-HDAg (27 kDa) in livers of two untreated and two treated mice (4 weeks) infected with either HBV/HDV-1a, HBV/HDV-1p, or HBV/HDV-3. The amount of human hepatocytes per liver specimen used to extract proteins was estimated by detecting human albumin (67 kDa). GAPDH, glyceraldehyde-3-phosphate dehydrogenase; HDAg, hepatitis D antigen; pegIFNα, pegylated interferon alpha.

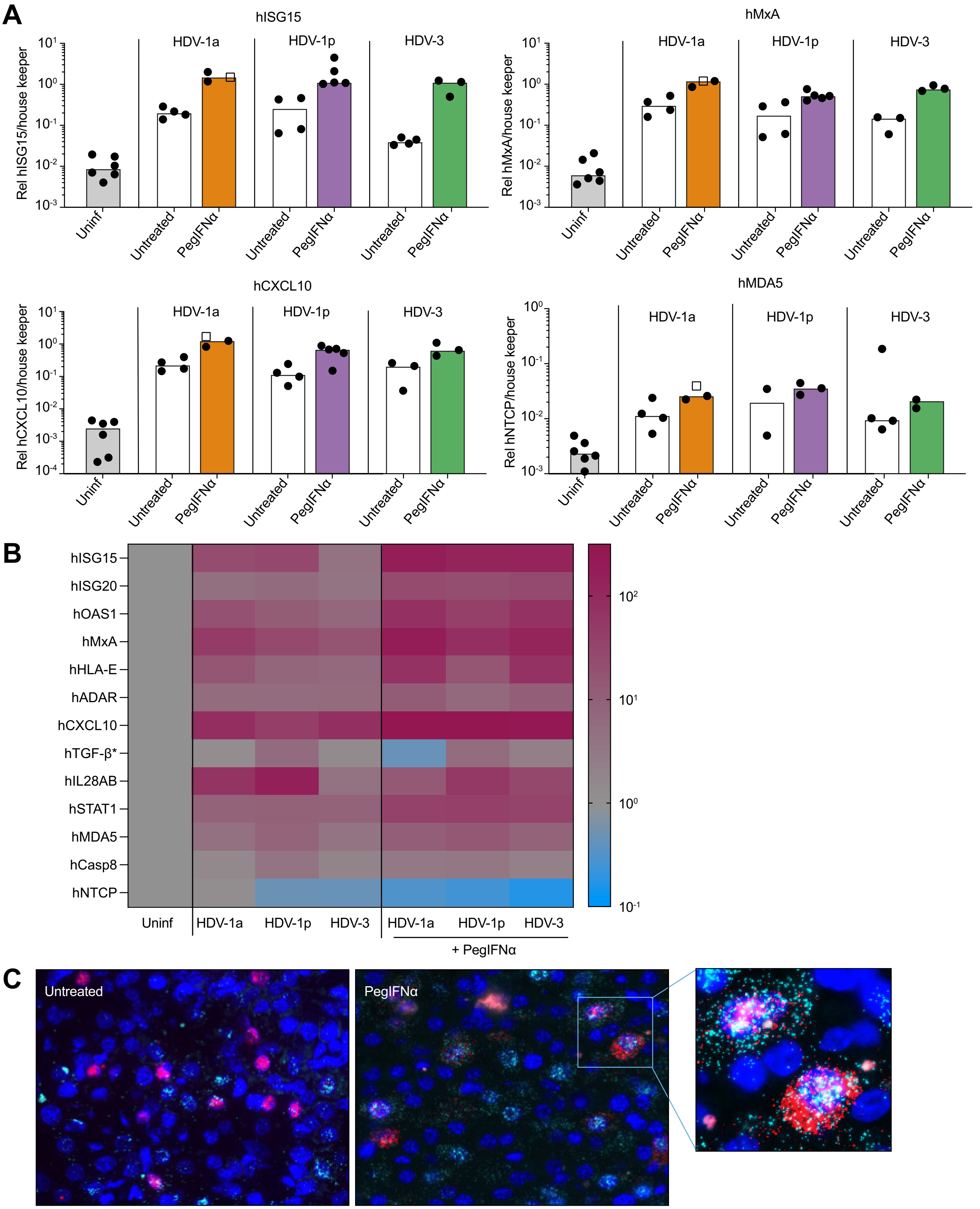

Fig. 5. PegIFNα-mediated hISG induction in HDV-1a, HBV/HDV-1p, and HBV/HDV-3 infected mice. (A) qPCR measurements of intrahepatic human hISG15, hMxA, hCXCL10, and hMDA5 mRNA levels in pegIFNα-treated or untreated infected mice compared with uninfected mice (n = 6). Bars show median level, black dots represent individual mice. HBV/HDV-1a-infected mice are shown as clear square or black dots when they were treated for 4 (n = 1) or 9 weeks (n = 2), respectively. (B) The heat map shows mRNA expression levels of different genes in untreated and treated mice of all groups as log induction compared with

treatment demonstrated lower numbers of HDAg-positive PHHs (HDV-1p: 4%, HDV-3: 30%) compared with untreated controls (HDV-1p: 42%, HDV-3: 63%) (Fig. 4A). This confirms that both HDV-1p and HDV-3 isolates are highly responsive to IFN treatment *in vivo* in mice stably infected with both HBV and HDV. In mice infected with the IFN-resistant HDV-1a strain, the amount of HDAg-positive PHHs appeared comparable between untreated controls (32%) and mice that received pegIFNα for 4 (33%) or 9 weeks (31%) (Fig. 4A).

RNA *in situ* hybridisation showed a clear reduction of genomic HDV RNA in treated HDV-1p and HDV-3 infected mice, while the amount of genomic HDV RNA positive PHHs remained similar in untreated and treated HDV-1a infected mouse livers (Fig. S2). Moreover, genomic and antigenomic HDV RNA levels determined by a strain-specific qPCR assay using biotinylated magnetic beads[17] revealed that pegIFNα reduced both HDV RNA forms in HDV-1p- and HDV-3-infected mice, but mice harbouring HDV-1a remained unaffected (Fig. 4B). Accordingly, levels of S- and L-HDAg decreased in HDV-1p and HDV-3 infected mouse livers receiving pegIFNα administration but remained comparable in untreated and treated HDV-1a infected mice (Fig. 4C).

IFNα responsiveness of HDV-1a and HDV-1p *in vitro* using PHHs isolated from infected mice

To further assess the different responsiveness of HDV-1a and HDV-1p to IFNα in a different experimental setting, PHHs were isolated from humanised mice that were either stably HBV/HDV-1a infected (1.8×10^8 HBV DNA copies/ml, 1.1×10^7 HDV RNA copies/ml) or HBV/HDV-1p infected (2.5×10^9 HBV DNA copies/ml, 1.5×10^7 HDV RNA copies/ml). Plated cells received IFNα for 2 weeks (Fig. 5A). In HDV-1p-infected PHHs, HDV RNA levels remained stable over time, whereas 7- and 14-day IFNα treatment decreased intracellular HDV RNA levels by 1.0-log (89%) and 1.6-log (98%), respectively, compared with untreated controls (Fig. 5B). IFNα also lowered HDV RNA levels in cell culture supernatant by 0.7-log (Fig. 5C), intracellular HBV pgRNA levels by 0.3-log (80%) (Fig. 5D). In contrast, but in line with the *in vivo* results, 2-week IFN treatment did not reduce HDV RNA levels in HDV-1a-infected PHHs (Fig. 5B), although intracellular pgRNA was reduced by 0.5-log (65%) (Fig. 5D). The total amount of cells, determined as ng RNA per well, was not substantially altered over time and by treatment (Fig. 5E).

PegIFNα-mediated human ISG induction in HBV/HDV-1a, HBV/HDV-1p, and HBV/HDV-3-infected mice

Since mice infected with HBV/HDV-1p or HBV/HDV-3 responded to IFNα treatment and mice infected with HBV and the HDV-1a strain were resistant to therapy, we investigated whether these differences in IFN responsiveness could be explained by the different ability of these viruses to induce intrinsic innate responses in infected hepatocytes. In line with a previous study,[19] human ISGs (*e.g.* hISG15, hMxA, hOAS1, hSTAT1), pattern recognition receptors (hMDA5), and chemokines (*e.g.* hCXCL10) were similarly and strongly upregulated (between 3- and 88-fold) upon HDV infection, regardless of whether the mice were infected with HBV/HDV-1a, HBV/HDV-1p, or HBV/HDV-3 (Fig. 6A and B, Table 2). PegIFNα treatment induced a further enhancement (between 2- and 29-fold) of human ISGs compared with untreated, infected mice (Fig. 6A and B, Table 2). Interestingly, despite the different HDV treatment outcomes, expression levels of analysed genes appeared comparable among treated animals, suggesting that virological differences, rather than the different enhancement of innate host responses, might be responsible for the antiviral effect of IFN *in vivo*. Furthermore, pegIFNα had no further effect on the expression of human cytokines (hIL28AB, human transforming growth factor-β [hTGF-β]) and on the apoptosis marker human caspase 8, but led to a substantial decrease (3- to 4-fold) of hNTCP expression levels (Fig. 6B, Table 2). Of note, hIL28AB and hTGF-β baseline expression levels are close to the detection limit in PHHs, therefore slight expression changes among groups must be interpreted cautiously.

Remarkably, simultaneous visualisation of HDV RNA and human ISGs by RNA ISH revealed that MxA-positive human hepatocytes were still expressing high levels of antigenomic HDV-1a RNA. These results demonstrate that the IFN-resistant HDV-1a isolate does not hamper the IFN-mediated induction of classical human ISGs at the single cell level (Fig. 6C).

Sequencing of the three distinct HDV genome strains from infected mice

Genome sequencing of intrahepatic HDV RNA in mice infected with HDV-1a, HDV-1p, or HDV-3 revealed that no mutations emerged after 4 or 9 weeks of pegIFNα treatment *in vivo* (data not shown). In addition, the occurrence of genomes encoding for the small (ACC) or large HDAg (ATC) (RNA editing at the amber/W site) remained similar in treated and untreated mice and was comparable between the different HDV isolates (Fig. S3A). The ribozyme site showed 100% identity between the two HDV-1 isolates and 89.6% identity between HDV-1 and HDV-3 (Fig. S3B). The open reading frame for the large HDAg (214 amino acids) showed 89.5% identity between HDV-1p and HDV-1a and 71.2% identity between HDV-1p and HDV-3, which translated into several amino acid changes (Fig. S3B and C). However, the amber/W site (aa196), the prenylation site (aa212), and other known post-translational modification sites remained fully conserved between the three different isolates (Fig. S3C). In line with Le Gal *et al.*,[41] the two HDV-1 strains showed a proline residue at the nuclear export site at position 205, whereas HDV-3 harbours a glycine, suggesting different virion secretion efficiencies across genotypes. Two unique differences that exclusively occurred in the IFN-resistant HDV-1a-isolate were detected in the coiled coil domain at position aa41 (leucine instead of isoleucine) and aa44 (isoleucine instead of leucine) (Fig. S3C). Although these are conservative mutations, we cannot exclude their impact on SUMOylation rates at position 42.

Discussion

In CHD, PegIFNα is commonly used as an off-label treatment, although its mode of action in HDV-infected hepatocytes is still unclear and responsiveness to IFN treatment remains limited.[16] Understanding HDV diversity and the mechanisms determining

uninfected controls. Baseline levels are grey, inductions red, and reductions blue. (C) RNA *in situ* hybridisation (RNAScope) staining of antigenomic HDV RNA (red) and hMxA (aqua) in HBV/HDV-1a infected mice. Nuclei are stained with DAPI (blue). CXCL10, C-X-C motif chemokine ligand 10; ISG, interferon stimulated gene; MDA5, melanoma differentiation-associated protein 5; MxA, myxovirus resistance gene A; pegIFNα, pegylated interferon alpha;

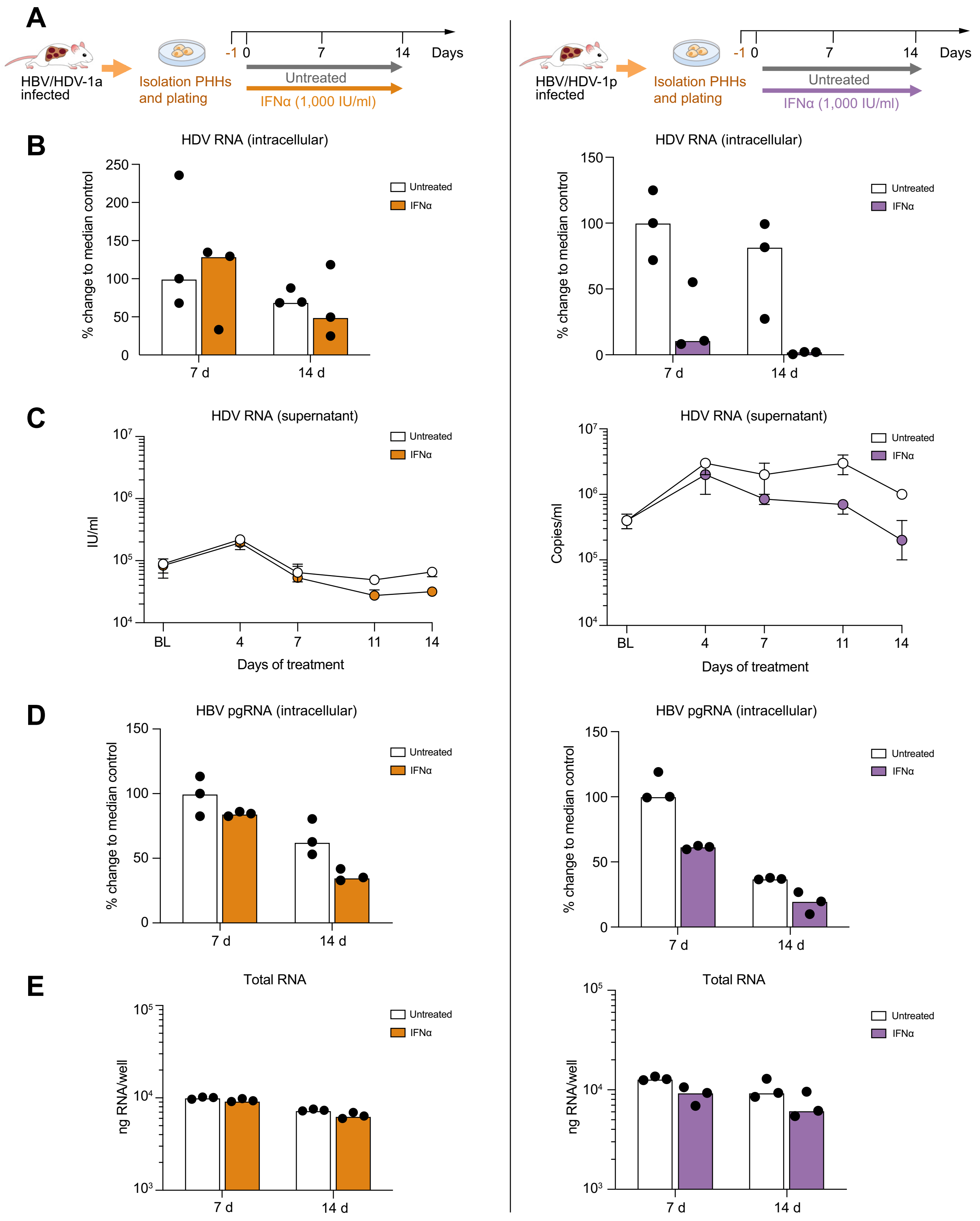

Fig. 6. IFNα treatment in HBV/HDV-1a- and HBV/HDV-1p-infected PHHs isolated from humanised mice. (A) Experimental design: PHHs were isolated from a stable HBV/HDV-1a- or HBV/HDV-1p-infected mouse and treated with IFNα. HDV RNA levels in PHHs (percent change from median controls at day 7) (B) and in cell culture supernatant (IU/ml; Baseline, day 4 and 7: n = 6; day 11 and 14: n = 3 for both groups) (C) of infected PHHs 7 or 14 days post IFNα treatment. Percent change of HBV pgRNA levels (D) and the amount of total RNA per well (E) in treated and untreated HBV/HDV-1a or HBV/HDV-1p infected PHHs. Bars show median

Table 2. mRNA expression of human genes.

	Uninfected	HDV-1a		HDV-1p		HDV-3		HDV-1a + pegIFNα		HDV-1p + pegIFNα		HDV-3 + pegIFNα	
Gene	Median rel expr	Median rel expr	Fold ind to uninf	Median rel expr	Fold ind to uninf	Median rel expr	Fold ind to uninf	Median rel expr	Fold ind to uninf	Median rel expr	Fold ind to uninf	Median rel expr	Fold ind to uninf
hISG15	8.70E-03	2.00E-01	23.0	2.55E-01	29.4	4.03E-02	4.6	1.50E+00	7.5	1.11E+00	4.3	1.15E+00	28.5
hISG20	5.72E-03	2.86E-02	5.0	3.55E-02	6.2	2.39E-02	4.2	1.44E-01	5.0	1.28E-01	3.6	1.52E-01	6.4
hOAS1	7.15E-03	1.38E-01	19.3	8.48E-02	11.9	5.08E-02	7.1	5.58E-01	4.0	2.87E-01	3.4	5.04E-01	9.9
hMxA	6.12E-03	3.04E-01	49.7	1.75E-01	28.6	1.06E-01	17.3	1.20E+00	3.9	5.20E-01	3.0	7.80E-01	7.4
hHLA-E	2.02E-02	3.18E-01	15.8	1.61E-01	8.0	1.33E-01	6.6	1.41E+00	4.4	3.30E-01	2.0	1.41E+00	10.6
hADAR	2.51E-02	1.48E-01	5.9	1.41E-01	5.6	1.59E-01	6.3	3.06E-01	2.1	1.62E-01	1.2	2.83E-01	1.8
hCXCL10	2.54E-03	2.23E-01	88.0	1.14E-01	45.0	2.10E-01	82.9	1.25E+00	5.6	6.77E-01	5.9	6.50E-01	3.1
hTGF-β	6.34E-04	7.64E-04	1.2	3.91E-03	6.2	8.81E-04	1.4	2.98E-04	0.4	3.81E-03	1.0	1.55E-03	1.8
hIL28AB	2.55E-05	1.72E-03	67.5	3.93E-03	154.3	1.17E-04	4.6	3.41E-04	0.2	1.38E-03	0.4	7.54E-04	6.5
hSTAT1	1.53E-02	1.38E-01	9.0	1.46E-01	9.5	1.40E-01	9.2	5.86E-01	4.2	6.04E-01	4.1	5.57E-01	4.0
hMDA5	2.35E-03	1.14E-02	4.8	1.98E-02	8.4	9.71E-03	4.1	2.60E-02	2.3	3.58E-02	1.8	2.15E-02	2.2
hCasp8	1.18E-03	1.72E-03	1.5	4.87E-03	4.1	2.26E-03	1.9	3.77E-03	2.2	4.01E-03	0.8	2.77E-03	1.2
hNTCP	3.12E-01	3.61E-01	1.2	1.54E-01	0.5	1.46E-01	0.5	9.89E-02	0.3	8.11E-02	0.5	5.46E-02	0.4

different antiviral responsiveness to treatment is paramount for improving therapeutic options. Experimental studies with the aim to unravel IFN MoA in HDV infection were hampered not only by the paucity of infection models available, but also by the limited availability of well-characterised HDV isolates. By chance, the first HDV-1 clone that was generated in 1988 (Kuo *et al.*,[29] herein HDV-1a), appeared IFN-resistant[28,29] both in transfected hepatoma cells and infected PHHs.[18,30,31] HDV was reduced only when IFNα was administered around HDV infection time,[18,30] indicating that IFNα mainly limited new infection events. These *in vitro* studies led to the general assumption that HDV is resistant to IFN treatment when infection is already established. However, we observed that human liver chimeric mice infected with an HDV-positive patient-derived serum responded to pegIFNα.[17] Interestingly, this HDV isolate (HDV-1p) was obtained from an individual who later achieved sustained HDV response upon pegIFNα treatment (Fig. 1 and Bockmann *et al.*[33]). To assess the IFN responsiveness of the most commonly used HDV-1a clone *in vivo*, in HBV/HDV infected human hepatocytes, and to compare these data with distinct HDV strains, we cloned this new HDV-1p isolate and also used an additional, genetically distant clone from an HDV-3 strain.[32]

In stable HBV/HDV-1p- and HBV/HDV-3-infected humanised mice, pegIFNα treatment clearly reduced HDV markers in serum and liver, including the levels of genomic and antigenomic HDV RNA, as well as S- and L-HDAg. In striking contrast, we did not detect any antiviral effect on HDV parameters when stable HBV/HDV-1a infected mice received pegIFNα, although all HBV markers, including HBsAg, were clearly reduced. Of note, the IFN dose commonly applied for studies in humanised mice is rather high (adapted to mouse metabolism) and, most importantly, it is given twice per week. Nevertheless, even extending treatment to 9 weeks did not alter HDV RNA levels or the number of HDV-positive human hepatocytes (as determined by immunofluorescence and RNA *in situ* hybridisation) when the HDV-1a strain was used. Notably, HBsAg is needed for the release of HDV particles and the IFN-mediated reduction of HBV serological markers, particularly those linked to cccDNA activity, was shown to be associated with virological responses in people with CHD.[42] Accordingly, IFN-mediated suppression of HBsAg production should also lower HDV RNA in serum. However, HBV infection and HBsAg levels are generally high in humanised mice and despite the decrease induced by IFN, HBsAg changes were not sufficient to induce substantial decrease of HDV-1a in IFN-treated mice. Our results indicate that also HDV viraemia changes need to be monitored to predict virological responses during IFN treatment.

Consistent with previous *in vitro* studies,[28,29] IFNα treatment of PHHs obtained from a stable HBV/HDV-1a-infected mouse had no effect on HDV RNA amounts. However, IFNα led to the reduction of HDV RNA levels in PHHs isolated from a stable HBV/HDV-1p-infected mouse. Because the virus strain was the only variable in these experimental settings (we used the same human hepatocyte donor and HBV inoculum), the sharp differences determined by using different HDV isolates demonstrate for the first time the existence of virus-derived differences. The key IFN-mediated antiviral mechanism acting in stably infected PHHs needs further investigation and the new genotype 1 HDV isolate could also serve for studies aiming at investigating HDV biology, as well as the antiviral activity of new compounds used alone and in combination with IFN.

As we did not measure induction of caspase 8 or detected substantial PHH loss *in vitro* and *in vivo* during treatment, the death of HDV-positive cells unlikely provides the main mechanism of intrahepatic HDV decline. Although we cannot rule out an increase of cellular stress induced both by HDV infection and interferon treatment, the strong HDV decrease determined in mice infected with HDV-1p or HDV-3 rather suggests that IFN inhibited HDV RNA synthesis and/or promoted its destabilisation.

The intrinsic innate responses of the hepatocytes are thought to be central to counteract HDV infection.[18,20] To

levels, black dots represent replicates. Blue bars: treated HDV-1a-infected PHHs, pink bars: treated HDV-1p-infected PHHs, grey bars: untreated PHHs. Curves display median levels and range. ADAR, adenosine deaminase; casp, caspase; CXCL10, C-X-C motif chemokine ligand 10; HLA, human leucocyte antigen; IFNα, interferon α; ISGs, interferon stimulated genes; MDA5, melanoma differentiation-associated protein 5; MxA, myxovirus resistance gene A; NTCP, sodium (Na^+) taurocholate co-transporting polypeptide; OAS1, 2′-5′-oligoadenylatsynthetase 1; PHHs, primary human hepatocytes; STAT1, signal transducers and activators of transcription 1; TGFβ, transforming growth factor-β.

investigate whether these three HDV strains may alter the ability of the human hepatocytes to sense the infection and to respond to IFN administration, we comparatively analysed classical human ISGs in infected and treated humanised mice.[19] Both the two HDV-1 isolates and the HDV-3 strain similarly enhanced human ISGs, including chemokines and pattern recognition receptors, compared with uninfected control mice. PegIFNα treatment increased the levels of human IFN signalling genes even further and – interestingly – in a comparable manner among all three HDV isolates used, irrespective of their responsiveness to treatment. Even at the single-cell level, HDV-1a-RNA-positive PHHs strongly co-expressed hMxA mRNA, suggesting that this IFN-resistant HDV-1a isolate does not hamper the broad IFN-mediated upregulation of intrinsic innate responses of human hepatocytes *in vivo*. Nevertheless, as we analysed a limited number of genes, we cannot exclude the possibility that other genes may be differentially expressed. Based on these results, it appears that the hepatocyte innate responses cannot be solely responsible for the strikingly different IFN responsiveness of distinct HDV isolates determined in stable HDV-infected cells, again hinting at the existence of virus-specific factors affecting the strength of IFN responsiveness. We did not identify clear mutations in the ribozyme and HDAg-coding region on the HDV genome or at the RNA editing site upon IFN treatment. Interestingly, all known post-translational modification sites in the HDAg open reading frame, including the prenylation site at position 212 and the nuclear localisation signal (aa-66-75) remained fully conserved among these three isolates. The two only differences that exclusively occurred in the IFN-resistant HDV-1a-isolate were detected in the coiled coil domain (leucine instead of isoleucine and *vice versa*) of the HDAg close to a SUMOylation site. However, leucine and isoleucine are both aliphatic, branched hydrophobic amino acids and these mutations may not influence SUMOylation rates at position 42. It remains to be investigated whether more complex sequence and conformational changes residing outside of the more conserved coding regions of HDV account for the intrinsic primary resistance to IFN determined with HDV-1a.

Recently, Zhang *et al.*[43] treated proliferating HDV-1a-infected hepatoma cells with IFNα and observed a strong block of cell division-mediated HDV spread,[43] suggesting that HDV RNA molecules can be targeted by intrinsic innate responses preferentially during cell division, a mechanism permitting NTCP-independent HDV spreading among daughter cells.[10] Experiments in human liver chimeric mice are usually started when human hepatocyte engraftment is completed and cell turnover is low.[9,10] Herein, we treated HBV/HDV-infected mice with IFN several weeks after PHH repopulation was accomplished and rates of PHH proliferation did not differ among mice infected with the distinct HDV isolates (Ki67-staining, data not shown). Although additional studies are required to explore the impact of IFN on distinct HDV strains during hepatocyte proliferation, cell turnover appears unlikely to explain the different IFN responses determined in the experimental setting used here. The ability of IFN to limit not only HDV infection events, but also to lower intrahepatic viral loads even in resting hepatocytes, would provide a rationale for the stronger anti-HDV effects determined in people with CHD receiving pegIFNα and the entry inhibitor bulevirtide in combination (MYR-203 clinical trial, NCT02637999).[44]

In conclusion, our study showed that two new patient-derived HDV isolates of genotype 1 and 3 respond to IFNα treatment in immune-deficient human liver chimeric mice. We also provide evidence that the commonly used HDV-1a isolate bears intrinsic capacities to resist IFNα treatment *in vivo*, which were not determined in two other patient-derived isolates. The existence of virus-specific determinants of IFNα responsiveness raises awareness of the need to use different HDV strains to evaluate virological and host-mediated mechanisms of IFN-responsiveness in HDV infection. The availability of new IFN-sensitive HDV strains could also contribute to the development of new therapies aiming at HDV cure.

Abbreviations

Actb, actin beta; ADAR, adenosine deaminase; ADF, adefovir; AG, antigenomic; BSA, bovine serum albumin; casp, caspase; CHD, chronic hepatitis D; CK18, cytokeratin 18; CXCL10, C-X-C motif chemokine ligand 10; Eef2, eukaryotic elongation factor; FCS, foetal calf serum; GAPDH, glyceraldehyde-3-phosphate dehydrogenase; hAAT, human alpha antitrypsin; HBsAg, hepatitis B virus surface antigen; HDAg, hepatitis delta antigen (S, small, L, large); HLA, human leucocyte antigen; HSA, uman serum albumin; IFNα, interferon α; ISGs, interferon stimulated genes; LAM, lamivudine; LLoD, lower limit of detection; Mavs, mitochondrial antiviral-signalling protein; MDA5, melanoma differentiation-associated protein 5; MoA, mode of action; MOI, multiplicity of infection; MxA, myxovirus resistance gene A; NTCP, sodium (Na+) taurocholate co-transporting polypeptide; NUCs, nucleos(t)ide analogues; OAS1, 2′-5′-oligoadenylatsynthetase 1; PEG, polyethylene glycol; pegIFNα, pegylated interferon alpha; PHHs, primary human hepatocytes; pgRNA, pregenomic RNA; Rig-I, retinoic acid-inducible gene I; RNP, ribonucleoprotein; qPCR, quantitative real time polymerase chain reaction; SCID, severe combined immunodeficiency; STAT1, signal transducers and activators of transcription 1; TGFβ, transforming growth factor-β; uPA, urokinase plasminogen activator; USG, uPA/SCID/beige/IL2RG-/-.

Financial support

The study was supported by the German Research Foundation (DFG) by a grant to MD and ML (SFB 841, A8), and to DG (GL595/9-1 and SFB1021, B08). MD and DG also received funding from the German Center for Infection Research (DZIF-BMBF; TTU-hepatitis 05.820; 05.822; 05.714). The National Reference Center for Hepatitis B Viruses and Hepatitis D Viruses is supported by the German Ministry of Health via the Robert Koch Institute. All funding sources supporting the work are acknowledged and authors have nothing to disclose.

Conflict of interest

The authors declare no competing interests.

Please refer to the accompanying ICMJE disclosure forms for further details.

Authors' contributions

Initiated and supervised the study: ML, MD. Designed experiments: ML, MD, KG. Generated chimeric mice: AV, LA, TV. Performed analyses and generated data: KG, LHen, PPG, JK, LHer. Performed HDV full genome sequencing: KG. Cloned the HDV-1p and tested their infectivity in HepG2^{hNTCP} cells: NG and DG. Provided clinical data: JHB, JP. Discussed the data: AV, JP, ML. Wrote the manuscript: KG, MD. Corrected the manuscript: NG, DG, AV, LA, PPG.

Data availability statement

All data, code, and materials used in the analysis are available upon reasonable request for collaborative studies regulated by materials/data transfer agreements (MTA/DTAs) to the corresponding author.

Acknowledgements
We are grateful to Roswitha Reusch and Sabrina Noster for excellent assistance with the mouse colony and Corinna Eggers and Martina Fahl for their great technical help. We thank John Taylor (Philadelphia, PA, USA) and John Casey (Washington, DC, USA) for fruitful discussions and for providing the HDV recombinant plasmid pSVL(D3) for HDV-1a, and pCMV3-Peru-1.2 for HDV-3, respectively. We also thank Camille Sureau (INTS, Paris, France) for providing infectious HDV-1a and HDV-3 particles and Jiabin Huang for his help in uploading the HDV-1p sequence to NCBI.

Supplementary data
Supplementary data to this article can be found online at https://doi.org/10.1016/j.jhepr.2023.100673.

References
Author names in bold designate shared co-first authorship

[1] Vlachogiannakos J, Papatheodoridis GV. New epidemiology of hepatitis delta. Liver Int 2020;40(Suppl 1):48–53.
[2] **Chen H-Y**, **Shen D-T**, **Ji D-Z**, Han P-C, Zhang W-M, Ma J-F, et al. Prevalence and burden of hepatitis D virus infection in the global population: a systematic review and meta-analysis. Gut 2019;68:512–521.
[3] Rizzetto M, Hamid S, Negro F. The changing context of hepatitis D. J Hepatol 2021;74:1200–1211.
[4] Koh C, Heller T, Glenn JS. Pathogenesis of and new therapies for hepatitis D. Gastroenterology 2019;156:461–476.e1.
[5] Dandri M, Volmari A, Lütgehetmann M. The hepatitis delta virus and chronic hepatitis D. J Hepatol 2022;77:1448–1450.
[6] Huang WH, Chen CW, Wu HL, Chen PJ. Post-translational modification of delta antigen of hepatitis D virus. Curr Top Microbiol Immunol 2006;307:91–112.
[7] Freitas N, Cunha C, Menne S, Gudima SO. Envelope proteins derived from naturally integrated hepatitis B virus DNA support assembly and release of infectious hepatitis delta virus particles. J Virol 2014;88:5742–5754.
[8] **Yan H**, **Zhong G**, Xu G, He W, Jing Z, Gao Z, et al. Sodium taurocholate cotransporting polypeptide is a functional receptor for human hepatitis B and D virus. Elife 2012;1:e00049.
[9] Allweiss L, Volz T, Giersch K, Kah J, Raffa G, Petersen J, et al. Proliferation of primary human hepatocytes and prevention of hepatitis B virus reinfection efficiently deplete nuclear cccDNA in vivo. Gut 2018;67:542–552.
[10] **Giersch K**, **Bhadra OD**, Volz T, Allweiss L, Riecken K, Fehse B, et al. Hepatitis delta virus persists during liver regeneration and is amplified through cell division both in vitro and in vivo. Gut 2019;68:150–157.
[11] **Roulot D**, **Brichler S**, Layese R, BenAbdesselam Z, Zoulim F, Thibault V, et al. Origin, HDV genotype and persistent viremia determine outcome and treatment response in patients with chronic hepatitis delta. J Hepatol 2020;73:1046–1062.
[12] Le Gal F, Gault E, Ripault M-P, Serpaggi J, Trinchet J-C, Gordien E, et al. Eighth major clade for hepatitis delta virus. Emerg Infect Dis 2006;12:1447–1450.
[13] Dény P. Hepatitis delta virus genetic variability: from genotypes I, II, III to eight major clades? Curr Top Microbiol Immunol 2006;307:151–171.
[14] Wedemeyer H, Schöneweis K, Bogomolov P, Blank A, Voronkova N, Stepanova T, et al. Safety and efficacy of bulevirtide in combination with tenofovir disoproxil fumarate in patients with hepatitis B virus and hepatitis D virus coinfection (MYR202): a multicentre, randomised, parallel-group, open-label, phase 2 trial. Lancet Infect Dis 2023;23:117–129.
[15] Caviglia GP, Rizzetto M. Treatment of hepatitis D: an unmet medical need. Clin Microbiol Infect 2020;26:824–827.
[16] **Urban S**, **Neumann-Haefelin C**, Lampertico P. Hepatitis D virus in 2021: virology, immunology and new treatment approaches for a difficult-to-treat disease. Gut 2021;70:1782–1794.
[17] Giersch K, Homs M, Volz T, Helbig M, Allweiss L, Lohse AW, et al. Both interferon alpha and lambda can reduce all intrahepatic HDV infection markers in HBV/HDV infected humanized mice. Sci Rep 2017;7:3757.
[18] Zhang Z, Filzmayer C, Ni Y, Sültmann H, Mutz P, Hiet M-S, et al. Hepatitis D virus replication is sensed by MDA5 and induces IFN-β/λ responses in hepatocytes. J Hepatol 2018;69:25–35.
[19] Giersch K, Allweiss L, Volz T, Helbig M, Bierwolf J, Lohse AW, et al. Hepatitis delta co-infection in humanized mice leads to pronounced induction of innate immune responses in comparison to HBV mono-infection. J Hepatol 2015;63:346–353.
[20] Dandri M, Bertoletti A, Lütgehetmann M. Innate immunity in hepatitis B and D virus infection: consequences for viral persistence, inflammation, and T cell recognition. Semin Immunopathol 2021;43:535–548.
[21] **Allweiss L**, **Giersch K**, Pirosu A, Volz T, Muench RC, Beran RK, et al. Therapeutic shutdown of HBV transcripts promotes reappearance of the SMC5/6 complex and silencing of the viral genome in vivo. Gut 2022;71:372–381.
[22] Zhang Z, Ni Y, Lempp FA, Walter L, Mutz P, Bartenschlager R, et al. Hepatitis D virus-induced interferon response and administered interferons control cell division-mediated virus spread. J Hepatol 2022;77:957–966.
[23] Sureau C, Taylor J, Chao M, Eichberg JW, Lanford RE. Cloned hepatitis delta virus cDNA is infectious in the chimpanzee. J Virol 1989;63:4292–4297.
[24] Makino S, Chang M-F, Shieh C-K, Kamahora T, Vannier DM, Govindarajan S, et al. Molecular cloning and sequencing of a human hepatitis delta (δ) virus RNA. Nature 1987;329:343–346.
[25] Chao YC, Lee CM, Tang HS, Govindarajan S, Lai MM. Molecular cloning and characterization of an isolate of hepatitis delta virus from Taiwan. Hepatology 1991;13:345–352.
[26] Ciccaglione AR, Rapicetta M, Fabiano A, Argentini C, Silvestro M, Giuseppetti R, et al. Chronic infection in woodchucks infected by a cloned hepatitis delta virus. Arch Virol Suppl 1993;8:15–21.
[27] Wang W, Lempp FA, Schlund F, Walter L, Decker CC, Zhang Z, et al. Assembly and infection efficacy of hepatitis B virus surface protein exchanges in 8 hepatitis D virus genotype isolates. J Hepatol 2021;75:311–323.
[28] Kuo MY, Chao M, Taylor J. Initiation of replication of the human hepatitis delta virus genome from cloned DNA: role of delta antigen. J Virol 1989;63:1945–1950.
[29] Kuo MY, Goldberg J, Coates L, Mason W, Gerin J, Taylor J. Molecular cloning of hepatitis delta virus RNA from an infected woodchuck liver: sequence, structure, and applications. J Virol 1988;62:1855–1861.
[30] Han Z, Nogusa S, Nicolas E, Balachandran S, Taylor J. Interferon impedes an early step of hepatitis delta virus infection. PLoS One 2011;6:e22415.
[31] Ilan Y, Klein A, Taylor J, Tur-Kaspa R. Resistance of hepatitis delta virus replication to interferon-alpha treatment in transfected human cells. J Infect Dis 1992;166:1164–1166.
[32] Casey JL, Brown TL, Colan EJ, Wignall FS, Gerin JL. A genotype of hepatitis D virus that occurs in northern South America. Proc Natl Acad Sci USA 1993;90:9016–9020.
[33] Bockmann J-H, Grube M, Hamed V, von Felden J, Landahl J, Wehmeyer M, et al. High rates of cirrhosis and severe clinical events in patients with HBV/HDV co-infection: longitudinal analysis of a German cohort. BMC Gastroenterol 2020;20:24.
[34] Casey JL, Gerin JL. Genotype-specific complementation of hepatitis delta virus RNA replication by hepatitis delta antigen. J Virol 1998;72:2806–2814.
[35] Rasche A, Lehmann F, König A, Goldmann N, Corman VM, Moreira-Soto A, et al. Highly diversified shrew hepatitis B viruses corroborate ancient origins and divergent infection patterns of mammalian hepadnaviruses. Proc Natl Acad Sci USA 2019;116:17007–17012.
[36] Nassal M. The arginine-rich domain of the hepatitis B virus core protein is required for pregenome encapsidation and productive viral positive-strand DNA synthesis but not for virus assembly. J Virol 1992;66:4107–4116.
[37] Sureau C, Guerra B, Lee H. The middle hepatitis B virus envelope protein is not necessary for infectivity of hepatitis delta virus. J Virol 1994;68:4063–4066.
[38] Langon T, Fillon S, Pichoud C, Hantz O, Trépo C, Kay A. Analysis of a hepatitis delta virus isolate from the Central African Republic. Res Virol 1998;149:171–185.
[39] **Lütgehetmann M**, **Mancke LV**, Volz T, Helbig M, Allweiss L, Bornscheuer T, et al. Humanized chimeric uPA mouse model for the study of hepatitis B and D virus interactions and preclinical drug evaluation. Hepatology 2012;55:685–694.
[40] Giersch K, Hermanussen L, Volz T, Volmari A, Allweiss L, Sureau C, et al. Strong replication interference between hepatitis delta viruses in human liver chimeric mice. Front Microbiol 2021;12:671466.
[41] Le Gal F, Brichler S, Drugan T, Alloui C, Roulot D, Pawlotsky J-M, et al. Genetic diversity and worldwide distribution of the deltavirus genus: a study of 2,152 clinical strains. Hepatology 2017;66:1826–1841.
[42] Sandmann L, Yurdaydin C, Deterding K, Heidrich B, Hardtke S, Lehmann P, et al. HBcrAg levels are associated with virological response to treatment with interferon in patients with hepatitis delta. Hepatol Commun 2022;6:480–495.
[43] Abstracts of the international liver congress june 23–26, 2021. Parallel sessions. J Hepatol 2021;75:S205–S293.
[44] Wedemeyer H, Schöneweis K, Bogomolov PO, Chulanov V, Stepanova T, Viacheslav M, et al. 48 weeks of high dose (10 mg) bulevirtide as monotherapy or with peginterferon alfa-2a in patients with chronic HBV/HDV co-infection. J Hepatol 2020;73:S52–S53.

Journal of Hepatology, Volume ■

Supplemental information

Strain-specific responsiveness of hepatitis D virus to interferon-alpha treatment

Katja Giersch, Paulina Perez-Gonzalez, Lennart Hendricks, Nora Goldmann, Jonathan Kolbe, Lennart Hermanussen, Jan-Hendrick Bockmann, Tassilo Volz, Annika Volmari, Lena Allweiss, Joerg Petersen, Dieter Glebe, Marc Lütgehetmann, and Maura Dandri

Strain-specific responsiveness of hepatitis D virus to interferon-alpha treatment

Katja Giersch, Paulina Perez-Gonzalez, Lennart Hendricks, Nora Goldmann, Jonathan Kolbe, Lennart Hermanussen, Jan-Hendrick Bockmann, Tassilo Volz, Annika Volmari, Lena Allweiss, Joerg Petersen, Dieter Glebe, Marc Lütgehetmann, Maura Dandri

Table of contents

Supplementary material and methods 2

Table S1 8

Fig. S1 9

Fig. S2. 10

Fig. S3. 11

Supplementary references........ **Error! Bookmark not defined.**

Supplementary material and methods

Generation of humanized USG mice. Human liver chimeric urokinase-type plasminogen activator (uPA)/severe combined immunodeficiency (SCID)/ beige/ interleukin-2 receptor gamma chain negative ($IL2RG^{-/-}$) mice (short USG mice) were generated by transplanting one million thawed cryo-preserved human hepatocytes into homozygous USG mice as previously reported [1]. Repopulation rates were estimated by determining human serum albumin (HSA) in mouse sera (ELISA; Bethyl Laboratories, Biomol GmbH, Hamburg, Germany) and human beta-globin in mouse liver DNA (qPCR; Taqman Gene Expression Assay Hs00758889_s1; Applied Biosystems, Carlsbad, USA). Animals displaying high levels of human chimerism (>2 mg/ml HSA in serum) were used for the study. All mice were sacrificed at the end of the experiment (at different time-points as indicated in the results), blood was collected and liver specimens were snap-frozen in chilled isopentane and cryo-conserved at -80°C for further histological and molecular analyses. Mice were maintained under specific pathogen free conditions in accordance with institutional guidelines under approved protocols. All animal experiments were conducted in accordance with the European Communities Council Directive (86/609/EEC) and were approved by the City of Hamburg, Germany.

Cell culture. PHHs were isolated either from one HBV/HDV-1a- or HBV/HDV-1p-infected human liver chimeric mouse and were seeded in 12- or 24-well plates for experiments. PHHs were maintained in William's E medium (Thermo Fisher, Waltham, MA, USA) supplemented with 10% Hyclone FBS (Thermo Fisher, Waltham, MA, USA), 1% GlutaMax (Thermo Fisher, Waltham, MA, USA), 4.7 µg/ml hydrocortisone, 0.1 µg/ml insulin, and 1.8% DMSO. Medium was changed 4 hours after plating and then twice a week. PHHs were maintained in culture for up to 15 days as indicated in the results.

$HepG2^{hNTCP}$ cells were maintained in DMEM medium (Thermo Fisher, Waltham, MA, USA) supplemented with 10% FCS and were seeded in 24-well plates or chamber slides for experiments. Medium was changed twice a week and $HepG2^{hNTCP}$ cells were split weekly (1:6)

before the experiments started. After infection 2% DMSO was added to the medium in order to minimize proliferation of HepG2^{hNTCP} cells. HepG2^{hNTCP} cells were maintained in culture for 7 days as indicated in the results.

Infection. HepG2^{hNTCP} cells were inoculated 24 hours after plating using HDV-1p, HDV-3 or HDV-1a (MOI=1-2) in FCS-free medium containing 4% PEG 8000 and 2% DMSO. 16-24 hours after inoculation the infection medium was removed and cells were washed twice with PBS. Infected cells were maintained until the end of the experiment as described above.

Chronic HBV-infected human liver chimeric USG mice (median viremia: 3x10^{8} copies HBV DNA/ml, HBV genotype D) were super-infected with 4x10^{6} HDV genome equivalents per mouse of HDV-1p containing cell culture supernatant. To establish an HBV/HDV-1a or HBV/HDV-3 infection in humanized USG mice, animals were co-infected using HBV (genotype D) and HDV-1a or HDV-3 containing cell culture supernatants (kindly provided by Dieter Glebe, Gießen, Germany and Camille Sureau, INTS, France, respectively) or passaged HBV/HDV-1A positive mouse sera (1x10^{7} HBV and HDV genome equivalents/mouse). The inoculum corresponded to a MOI of 0.1-0.3 by estimating an average of 3x10^{7} human hepatocytes per mouse liver [2].

Virological measurements in USG mice and PHHs. Viral DNA and RNA was extracted from 200 μl cell culture supernatant or 5 μl mouse serum using the QiAmp MinElute Virus Spin kit (Qiagen, Hilden, Germany) according to the manufacturer's instructions. At the end of the experiments, infected PHHs and mouse livers were collected and intracellular viral DNA and RNA was isolated using the MasterPure™ Complete DNA and RNA Purification Kit (Epicentre, Wisconsin, USA) and the Qiagen RNeasy Mini Kit, respectively.

HDV RNA levels in cell culture supernatant, cells, mouse serum and liver were determined by reverse transcription and qPCR using the ABI Fast 1-Step Virus Master (Applied Biosystems, Foster City, CA, USA) and HDV Taqman primers and probes on an ABI Viia7 (Applied Biosystems) as previously described [3]. HDV-specific primers and probes recognized all HDV

genotypes and HDV-1 strains [4]. HDV RNA levels in cell culture supernatant were also measured using the cobas6800 automated system (Roche, Basel, Switzerland) as previously described [5]. HBV DNA levels in cell culture supernatant and mouse serum as well as HBV pregenomic (pg) RNA levels in cells and mouse liver were determined by qPCR using specific primers and probe (Taqman Gene Expression Assay Pa03453406_s1, Applied Biosystems and [6]) under conditions previously described [3]. Known amounts of an HDV- or HBV-containing plasmid were used as standard for HDV RNA and HBV DNA quantification in serum and cell culture supernatants. Steady-state levels of intracellular viral RNA and DNA amounts were normalized to the median of human specific hGAPDH and hRPL30 (Taqman Gene Expression Assay Hs99999905_m1 and Hs00265497_m1, Applied Biosystems) using the $\Delta\Delta$ct method.

Genomic and antigenomic HDV RNA qPCR assay. Genomic and antigenomic HDV RNA were determined using a biotinylated magnetic beads based qPCR assay as described previously [7]. In brief, RNA extracted from 1 µl mouse liver was reverse transcribed with 0.5 µM of a biotinylated HDV specific forward primer (biotin-GCGCCGGCYGGGCAAC) for genomic HDV RNA or a biotinylated HDV specific reverse primer (biotin-TTCCTCTTCGGGTCGGCATG) for antigenomic HDV RNA detection and the ABI Fast 1-Step Virus Master (Applied Biosystems, Carlsbad, USA). Biotinylated cDNA was purified with the MinElute PCR Purification Kit (Qiagen, Hilden, Germany) and isolated with dynabeads specifically interacting with biotin (Dynal Kilobase Binder Kit, Invitrogen, Darmstadt, Germany) following the manufacturer’s instructions. For qPCR 1 µl of purified biotinylated cDNA bound to dynabeads, HDV specific primers and probes [4] and the ABI Fast Advanced Master (Applied Biosystems, Carlsbad, USA) were used. The median of two human-specific housekeeping genes (hGAPDH, Hs99999905_m1, and hRPL30, Hs00265497_m1, Applied Biosystems) were used for normalization.

HDAg Western blot. Western blot of mouse or patient liver tissue was performed as previously described [8]. In brief, protein lysates were obtained by extracting tissue with T-Per Tissue Protein Extraction Reagent (Pierce, Rockford, United States) supplemented with protease and phosphatase inhibitors. Protein content was measured by Pierce BCA Protein Assay Kit (Thermo Scientific, Rockford, United States). Proteins were resolved on 12% Mini-PROTEAN TGX Precast Gels (Bio-Rad, Feldkirchen, Germany) and blotted on nitrocellulose membranes (0.2 µm pore size; GE Healthcare, Buckinghamshire, UK). S-HDAg and L-HDAg were detected using a rabbit anti-Delta antibody (1:1,000) (kindly provided by John Taylor, Philadelphia, PA USA). Amounts of human hepatocytes were determined by using a mouse anti-human-albumin antibody (1:200,000) (#A6684; Sigma-Aldrich, St. Louis, Missouri, USA). Signals were visualised with Pierce ECL Western Blotting Substrate (Thermo Fisher) and the Fusion FX Imager (Vilber).

Sequencing. For HDV genome sequencing, serum RNA from HBV/HDV-1p- or HDV-1A-infected mice was extracted as described above and cDNA was synthesized with the Transcriptor First Strand cDNA Synthesis Kit (Roche, Basel, Switzerland) using random hexamer primer according to the manufacturer’s instructions. To generate the full genome sequence of HDV-1p overlapping PCR fragments were generated using 5 HDV-specific primer pairs [9] and a Red-Taq Polymerase (Sigma-Aldrich, St. Louis, USA) under conditions described previously [9]. To analyze the occurrence of mutations in treated HDV-1p- or HDV-1A-infected mice, the HDV-specific primer pairs R1 and R2 [10] were used as described previously [11]. PCR product length was analyzed on a 0.8% agarose gel and DNA fragments were purified with the MinElute PCR Purification Kit (Qiagen) as recommended by the manufacturer. The forward and reverse strand was sequenced with Sanger sequencing (Mix2seq kit) by Eurofins Genomics (Ebersberg, Germany) and data was analyzed using Geneious R6 (BioMatters, Auckland, New Zealand).

Expression of human interferon stimulated genes (ISGs). To determine intracellular expression levels of human interferon stimulated genes (ISGs) in USG mice, intracellular RNA was extracted as described above and cDNA was synthesized with the Transcriptor First Strand cDNA Synthesis Kit (Roche, Basel, Switzerland) using oligo-dT primer according to the manufacturer's instructions. qPCR was performed with the ABI Fast Advanced Master (Applied Biosystems) in an ABI Viia7 (Applied Biosystems) and by using Taqman Gene Expression Assays from Applied Biosystems containing human-specific primers and probe, which do not cross-react with murine genes (**suppl. table 1**). The human housekeeping genes hGAPDH (Hs99999905_m1) and hRPL30 (Hs00265497_m1) were used for normalization.

Immune histology. Paraffin-embedded patient biopsies were deparaffinized with xylene, rehydrated with ethanol and boiled in citrate buffer (antigen retrieval). After endogenous protease and protein block biopsies were stained using a rabbit anti-Delta antibody (1:10,000) (kindly provided by John Taylor, Philadelphia, PA USA) at 4 °C overnight. Specific signals were visualized with Dako Envision+ system HRP-labeled polymer anti-rabbit and Dako Liquid DAB+ Substrate Chromogen System (Dako, Glostrup, Denmark). Counterstaining was performed with Mayer's Hematoxylin (Sigma-Aldrich, St. Louis, USA) and slides were dehydrated and mounted. Stained sections were analyzed by brightfield microscopy (Biorevo BZ-9000, Keyence).

Immunofluorescence (IF) staining. Cryostat sections of humanized USG mouse livers were fixed with acetone and stained as previously described [1]. Tissue was incubated with mouse anti-CK18 (1:400, Dako, Glostrup, Denmark), rabbit anti-HBcAg (1:2,000, Dako) and human anti-Delta (anti-HDAg-positive human serum, 1:8,000) primary antibodies at 4 °C overnight. Specific signals were visualized with Alexa 488-, or 555-labeled secondary antibodies (Invitrogen, Darmstadt, Germany). Nuclei were stained with Hoechst 33258 (1:20,000 diluted, Invitrogen). Stained sections were then mounted with fluorescent mounting medium (Dako) and analyzed with the fluorescence microscope BZ8710 (Keyence, Osaka, Japan) using the

same settings for the different experimental groups. The percentage of HDAg-positive human hepatocytes was counted manually and by using 3 visual fields (displaying an average of 300-500 human hepatocytes) per mouse liver.

RNA in situ hybridization (RNAScope). RNA *in situ* hybridization was performed on paraformaldehyde-fixed, cryo-preserved mouse liver sections using the RNAScope Fluorescent Multiplex Kit (Advanced Cell Diagnostics, ACD, Hayward, CA, USA) according to the manufacturer's instructions and as previously described [12]. Briefly, liver sections were fixed with 4% paraformaldehyde (PFA), dehydrated with ethanol and pretreated with Pretreat 4 (Pretreatment Kit, ACD) for 30 min. Liver sections were then incubated with RNAScope target probes, which specifically bind HDV-1p genomic (G) HDV RNA (ACD assay number: 484611), HDV-1A genomic (G) HDV RNA (478131), HDV-1A antigenomic (AG) HDV RNA (475311) or hMxA (403831-C3) for 2 h at 40°C (HybEZ oven, ACD). DAPI staining was performed to visualize nuclei. Stained sections were analyzed by fluorescence microscopy (Biorevo BZ-9000, Keyence) using a 60×/1.40 NA oil objective. Merged z stack images were prepared using the same settings for all groups.

Software. Graphs were created with GraphPad Prism (Version 9.3). The graphical abstract was designed with BioRender.

Gene	Assay number (Applied Biosystems)
hISG15	Hs00192713_m1
hISG20	Hs00158122_m1
hOAS1	Hs00973637_m1
hMxA	Hs00895608_m1
hHLA-E	Hs03045171_m1
hADAR	Hs01017595_g1
hCXCL10	Hs00171042_m1
hTGF-β	Hs00171257_m1
hIL28AB	Hs04193049_gH
hSTAT1	Hs01013989_m1
hMDA5	Hs01070332_m1
hCasp8	Hs01018151_m1
hNTCP	Hs00914889_m1

Table S1. Taqman Gene Expression Assays containing human-specific primers and probe. The table shows target genes and assay numbers from Applied Biosystems.

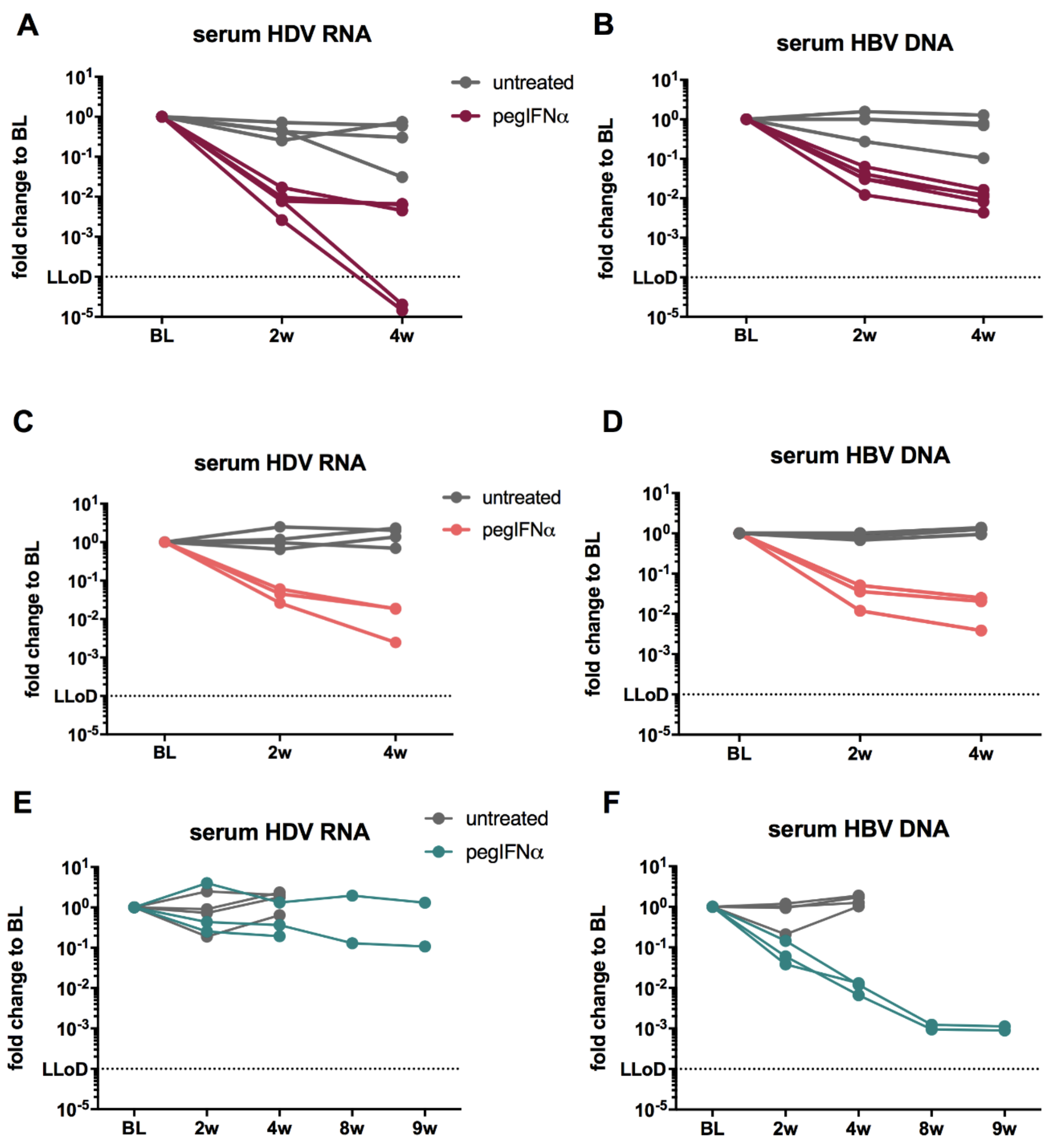

Fig. S1. HDV and HBV viremia in individual mice. Serum HDV RNA (**A, C, E**) and HBV DNA (**B, D, F**) levels depicted as fold change from baseline of individual HBV/HDV-1p- (**A, B**), HBV/HDV-3-infected mice (**C, D**) HBV/HDV-1a- (**E, F**), which were treated with pegIFNα for 4 or 9 weeks or remained untreated.

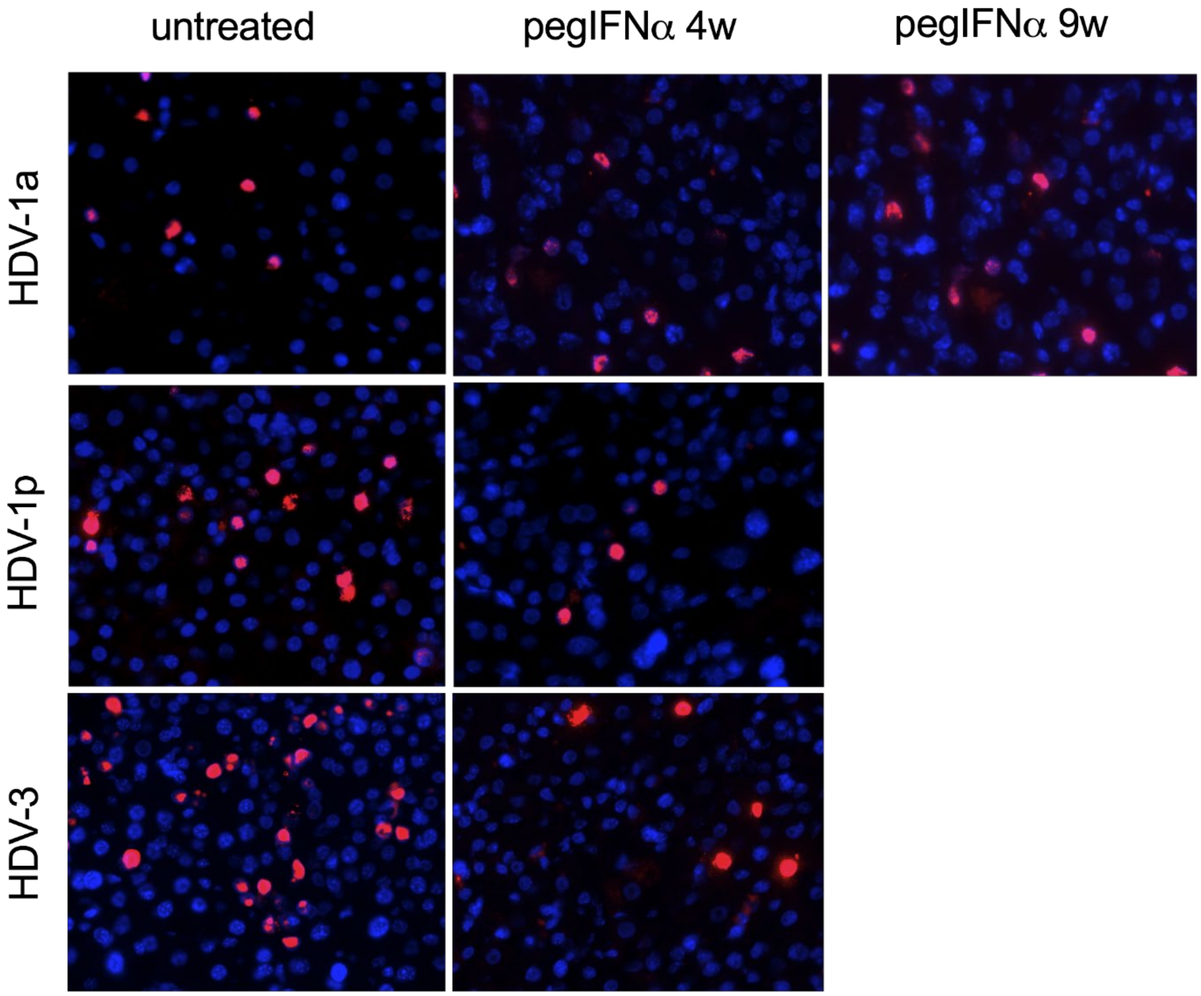

Fig. S2. RNA in situ hybridization in pegIFNα treated mice. B) RNA in situ hybridization of genomic HDV RNA (red) in mouse livers of all groups. Nuclei are stained with Dapi.

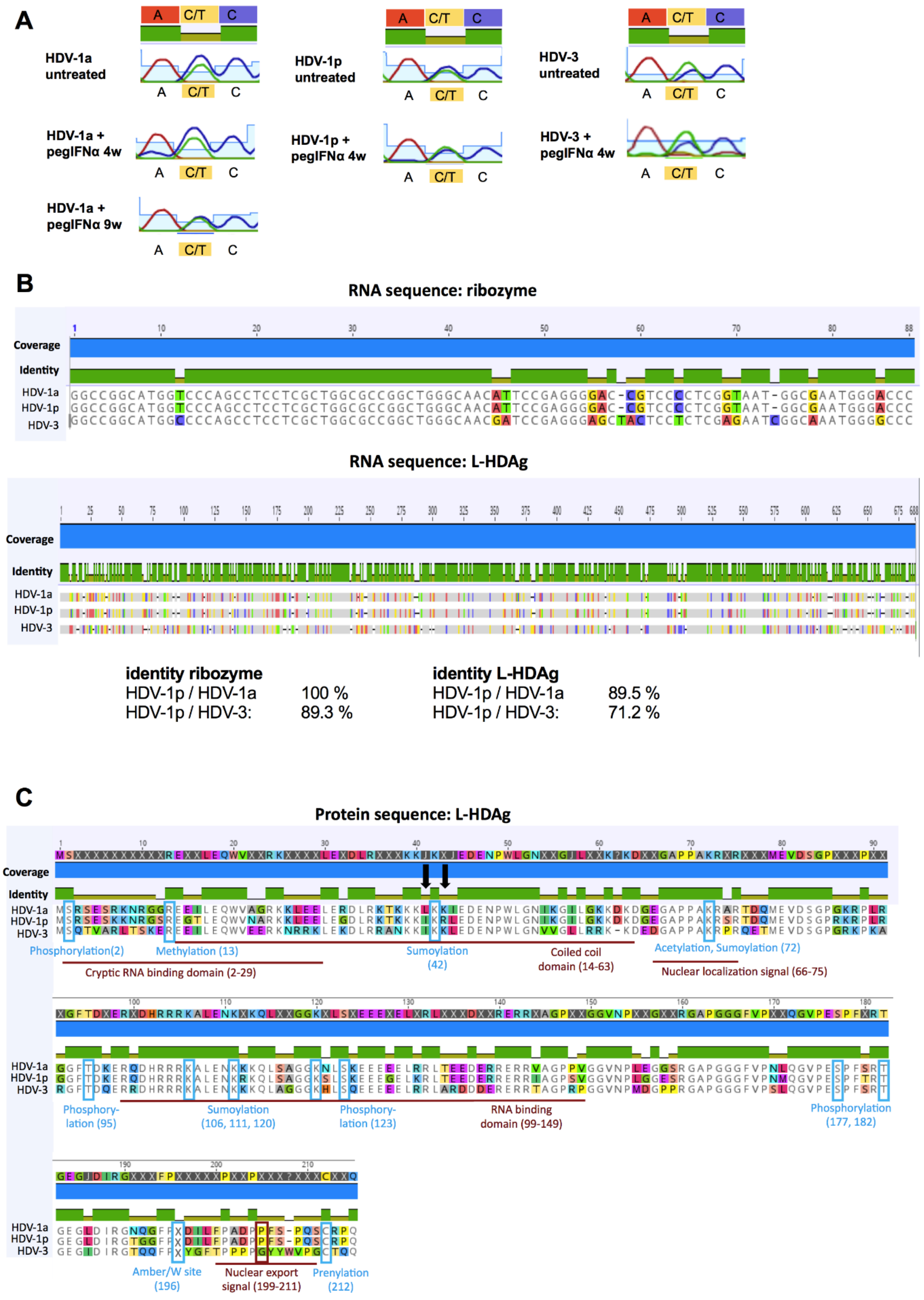

Fig. S3. HDV RNA genome sequencing in HDV-1a, HDV-1p- and HDV-3-infected mice. A) HDV RNA genome sequence of the amber/W site (RNA editing) in untreated and pegIFNα-treated HBV/HDV-1a, HBV/HDV-1p- or HBV/HDV-3-infected mice. ATC (stop codon) encodes

for the S-HDAg, ACC encodes for the L-HDAg. **B)** HDV-1a, HDV-1p and HDV-3 genome sequencing of the open reading frame encoding for the large HDAg (645 nucleotides). **C**) Alignment of the protein sequence of the large HDAg (214 amino acids) of HDV-1a, HDV-1p and HDV-3. Unique differences that exclusively occur in the IFN-resistant HDV-1A-isolate are marked with a black arrow (aa-41, aa-44).

Supplementary references

1. **Lütgehetmann, M.; Mancke**, L.V.; Volz, T.; Helbig, M.; Allweiss, L.; Bornscheuer, T.; Pollok, J.M.; Lohse, A.W.; Petersen, J.; Urban, S.; et al. Humanized Chimeric UPA Mouse Model for the Study of Hepatitis B and D Virus Interactions and Preclinical Drug Evaluation. *Hepatology* **2012**, *55*, 685–694, doi:10.1002/hep.24758.

2. Dandri, M.; Murray, J.M.; Lutgehetmann, M.; Volz, T.; Lohse, A.W.; Petersen, J. Virion Half-Life in Chronic Hepatitis B Infection Is Strongly Correlated with Levels of Viremia. *Hepatology* **2008**, *48*, 1079–1086, doi:10.1002/hep.22469.

3. **Giersch, K.; Bhadra, O.D.**; Volz, T.; Allweiss, L.; Riecken, K.; Fehse, B.; Lohse, A.W.; Petersen, J.; Sureau, C.; Urban, S.; et al. Hepatitis Delta Virus Persists during Liver Regeneration and Is Amplified through Cell Division Both in Vitro and in Vivo. *Gut* **2019**, *68*, 150–157, doi:10.1136/gutjnl-2017-314713.

4. Ferns, R.B.; Nastouli, E.; Garson, J.A. Quantitation of Hepatitis Delta Virus Using a Single-Step Internally Controlled Real-Time RT-QPCR and a Full-Length Genomic RNA Calibration Standard. *J Virol Methods* **2012**, *179*, 189–194, doi:10.1016/j.jviromet.2011.11.001.

5. Pflüger, L.S.; Nörz, D.; Volz, T.; Giersch, K.; Giese, A.; Goldmann, N.; Glebe, D.; Bockmann, J.-H.; Pfefferle, S.; Dandri, M.; et al. Clinical Establishment of a Laboratory Developed Quantitative HDV PCR Assay on the Cobas6800 High-Throughput System. *JHEP Rep* **2021**, *3*, 100356, doi:10.1016/j.jhepr.2021.100356.

6. Malmström, S.; Larsson, S.B.; Hannoun, C.; Lindh, M. Hepatitis B Viral DNA Decline at Loss of HBeAg Is Mainly Explained by Reduced CccDNA Load--down-Regulated Transcription of PgRNA Has Limited Impact. *PLoS One* **2012**, *7*, e36349, doi:10.1371/journal.pone.0036349.

7. Giersch, K.; Homs, M.; Volz, T.; Helbig, M.; Allweiss, L.; Lohse, A.W.; Petersen, J.; Buti, M.; Pollicino, T.; Sureau, C.; et al. Both Interferon Alpha and Lambda Can Reduce All Intrahepatic HDV Infection Markers in HBV/HDV Infected Humanized Mice. *Sci Rep* **2017**, *7*, 3757, doi:10.1038/s41598-017-03946-9.

8. **Giersch, K.; Hermanussen**, L.; Volz, T.; Volmari, A.; Allweiss, L.; Sureau, C.; Casey, J.; Huang, J.; Fischer, N.; Lütgehetmann, M.; et al. Strong Replication Interference Between Hepatitis Delta Viruses in Human Liver Chimeric Mice. *Front Microbiol* **2021**, *12*, 671466, doi:10.3389/fmicb.2021.671466.

9. Pyne, M.T.; Mallory, M.A.; Xie, H.B.; Mei, Y.; Schlaberg, R.; Hillyard, D.R. Sequencing of

the Hepatitis D Virus RNA WHO International Standard. *J Clin Virol* **2017**, *90*, 52–56, doi:10.1016/j.jcv.2017.03.009.

10. Ivaniushina, V.; Radjef, N.; Alexeeva, M.; Gault, E.; Semenov, S.; Salhi, M.; Kiselev, O.; Dény, P. 2001 Hepatitis Delta Virus Genotypes I and II Cocirculate in an Endemic Area of Yakutia, Russia. *Journal of General Virology 82*, 2709–2718, doi:10.1099/0022-1317-82-11-2709.

11. **Giersch, K.; Helbig**, M.; Volz, T.; Allweiss, L.; Mancke, L.V.; Lohse, A.W.; Polywka, S.; Pollok, J.M.; Petersen, J.; Taylor, J.; et al. Persistent Hepatitis D Virus Mono-Infection in Humanized Mice Is Efficiently Converted by Hepatitis B Virus to a Productive Co-Infection. *J Hepatol* **2014**, *60*, 538–544, doi:10.1016/j.jhep.2013.11.010.

12. **Allweiss, L.; Gass**, S.; Giersch, K.; Groth, A.; Kah, J.; Volz, T.; Rapp, G.; Schöbel, A.; Lohse, A.W.; Polywka, S.; et al. Human Liver Chimeric Mice as a New Model of Chronic Hepatitis E Virus Infection and Preclinical Drug Evaluation. *J Hepatol* **2016**, *64*, 1033–1040, doi:10.1016/j.jhep.2016.01.011.
